# Multifaceted Roles of Chemokines and Chemokine Receptors in Tumor Immunity

**DOI:** 10.3390/cancers13236132

**Published:** 2021-12-06

**Authors:** Kazuhiko Matsuo, Osamu Yoshie, Takashi Nakayama

**Affiliations:** 1 Division of Chemotherapy, Faculty of Pharmacy, Kindai University, Osaka 577-8502, Japan; matsuo@phar.kindai.ac.jp; 2 Health and Kampo Institute, Murasakiyama, Izumi, Sendai 981-3205, Japan; qqud7p89k@siren.ocn.ne.jp; 3 Kindai University, Osaka 577-8502, Japan; 4 Aoinosono Sendai Izumi Long-Term Health Care Facility, Izumi, Sendai 981-3126, Japan

**Keywords:** chemokine, chemokine receptor, Th1, Th2, Th17, regulatory T, dendritic cell, macrophage, neutrophil, tumor microenvironment

## Abstract

**Simple Summary:**

Various immune cells are involved in host immune responses to cancer. T-helper (Th) 1 cells, cytotoxic CD8^+^ T cells, and natural killer cells are the major effector cells in anti-tumor immunity, whereas cells such as regulatory T cells and myeloid-derived suppressor cells are negatively involved in anti-tumor immunity. Th2 cells and Th17 cells have been shown to have both pro-tumor and anti-tumor activities. The migratory properties of various immune cells are essential for their function and critically regulated by the chemokine superfamily. In this review, we summarize the roles of various immune cells in tumor immunity and their migratory regulation by the chemokine superfamily. We also assess the therapeutic possibilities of targeting chemokines and chemokine receptors in cancer immunotherapy.

**Abstract:**

Various immune cells are involved in host tumor immune responses. In particular, there are many T cell subsets with different roles in tumor immunity. T-helper (Th) 1 cells are involved in cellular immunity and thus play the major role in host anti-tumor immunity by inducing and activating cytotoxic T lymphocytes (CTLs). On the other hand, Th2 cells are involved in humoral immunity and suppressive to Th1 responses. Regulatory T (Treg) cells negatively regulate immune responses and contribute to immune evasion of tumor cells. Th17 cells are involved in inflammatory responses and may play a role in tumor progression. However, recent studies have also shown that Th17 cells are capable of directly inducting CTLs and thus may promote anti-tumor immunity. Besides these T cell subsets, there are many other innate immune cells such as dendritic cells (DCs), natural killer (NK) cells, and myeloid-derived suppressor cells (MDSCs) that are involved in host immune responses to cancer. The migratory properties of various immune cells are critical for their functions and largely regulated by the chemokine superfamily. Thus, chemokines and chemokine receptors play vital roles in the orchestration of host immune responses to cancer. In this review, we overview the various immune cells involved in host responses to cancer and their migratory properties regulated by the chemokine superfamily. Understanding the roles of chemokines and chemokine receptors in host immune responses to cancer may provide new therapeutic opportunities for cancer immunotherapy.

## 1. Introduction

Tumor immunity is initiated by the recognition of tumor antigens by the immune system [1]. Antigen priming and effector cell differentiation are regulated by complex processes involving various cell populations, cytokines, and chemokines [1,2,3]. While CD4^+^ helper T cells are known to orchestrate immune responses, CD4^+^ T cells are heterogenous and composed of various functional subsets including T-helper (Th) 1 cells, Th2 cells, Th17 cells, and regulatory T (Treg) cells [4,5]. These T cell subsets are characterized by the secretion of signature cytokines and differentially involved in tumor immunity [4,5]. Furthermore, CD8^+^ cytotoxic lymphocytes (CTLs) and natural killer (NK) cells play direct roles in the elimination of tumor cells. Tumor tissues are also highly enriched with innate immune cells such as myeloid-derived suppressor cells (MDSCs), tumor-associated macrophages (TAMs), and tumor-associated neutrophils (TANs) [6,7]. These various immune cells are known to express characteristic chemokine receptors and are elaborately regulated in their migration and tissue localization by respective chemokine ligands [8,9,10]. Accordingly, some chemokines and their receptors may be involved in the elimination of tumor cells by recruiting anti-tumor effector cells, whereas others may promote tumor progression by attracting immunosuppressive cells. In addition, some chemokines and their receptors are known to play critical roles in the interactions of dendric cells (DCs) and T cells [11,12,13,14]. Thus, the chemokine superfamily has a multifaceted role in host tumor immunity. In this review, we overview the roles of various immune cells and the chemokine superfamily in host immune responses to tumor cells in order to assess the possibility of targeting chemokines and chemokine receptors for cancer immunotherapy.

## 2. Induction of Tumor-Specific T Cell Responses

**Figure 1:**
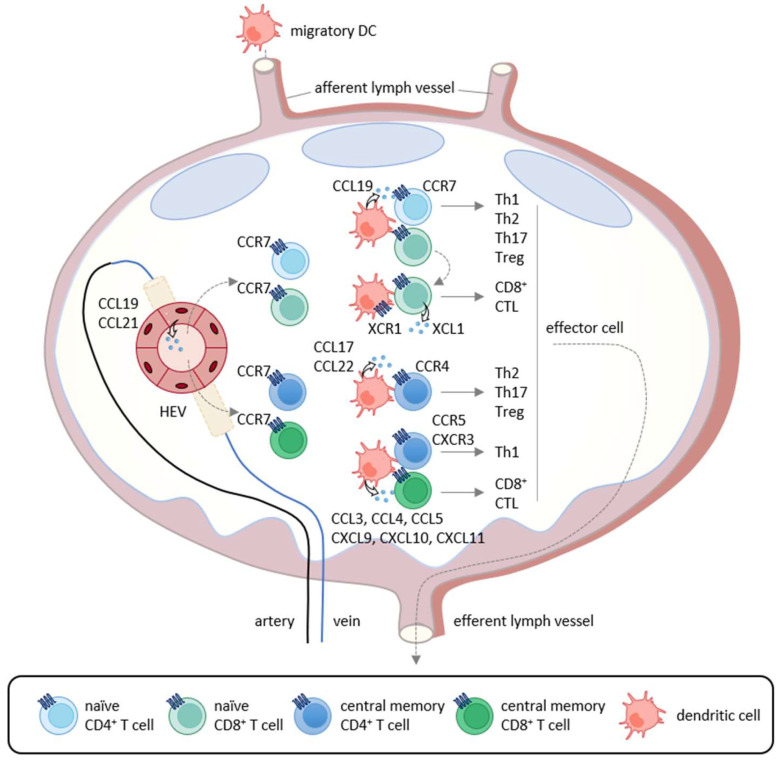
Chemokine-mediated T cell immune responses in the lymph node. In primary immune responses, antigen-captured DCs migrate into the draining lymph nodes via afferent lymphatics using the CCL19/CCL21-CCR7 axis. In the lymph nodes, mature DCs produce CCL19 and interact with recirculating CCR7-expressing naïve CD4^+^ T cells that home into the lymph nodes via the high endothelial venules (HEVs). If stimulated by cognate antigenic peptides presented by mature DCs, naïve T cells differentiate into Th1 cells, Th2 cells, Th17 cells, and Treg cells according to the local cytokine milieu.

T cell-mediated anti-tumor immunity is thought to be achieved by a multistep process called the cancer-immunity cycle [1]. It includes the following seven steps: (1) release of cancer antigens, (2) cancer antigen presentation, (3) priming and activation, (4) trafficking of T cells to tumors, (5) infiltration of T cells into tumors, (6) recognition of cancer cells by T cells, and (7) killing of cancer cells. Thus, the cycle is initiated by the uptake of tumor antigens, including tumor-associated antigens and neoantigens, by DCs, the professional antigen-presenting cells. Tumor antigen-captured DCs then migrate into the draining lymph nodes where recirculating naïve T cells and memory T cells scan antigenic peptides presented by DCs in association with class I and class II major histocompatibility complex (MHC) molecules (Figure 1). The CCL19/CCL21-CCR7 axis is known to play a pivotal role in the migratory activities of DCs and recirculating T cells [1,15]. While immature DCs in peripheral tissues dominantly express CCR6, the surface expression of CCR7 is upregulated upon antigen-loading and DC maturation [1,15]. Since CCL21 is abundantly produced by lymphatic vessels, CCR7-expressing DCs initiate trans-lymphatic migration and home into T cell areas of the draining lymph nodes where matured DCs start producing CCL19 (Figure 1) [3,16,17]. The lymph nodes also have unique vascular structures called high endothelial venules (HEVs), which produce CCL21 and function as the gateways for recirculating naïve T cells and memory T cells that commonly express CCR7 (Figure 1) [18,19]. In addition, although CCL19 is not produced by HEVs, it is displayed on the luminal surfaces of HEVs by transcytosis [20]. After homing into the lymph nodes, naïve T cells and memory T cells further migrate toward CCL19-producing mature DCs localized in the T cell areas [1,15]. Upon encounter with cognate antigenic peptides presented by mature DCs, antigen-specific naïve T cells proliferate and differentiate into various effector T cell subsets in accordance with the local cytokine milieu, whereas memory T cells start rapid expansions for recall immune responses [21]. Furthermore, conventional DCs have two subtypes known as type 1 (cDC1s) and type 2 (cDC2s) [22]. It is now known that cDC1s preferentially induce the differentiation of naïve CD4^+^ T cells and CD8^+^ T cells into Th1 cells and CTLs, respectively [3,16,17], whereas cDC2s preferentially induce the differentiation of naïve CD4^+^ T cells into Th2 cells and Th17 cells (Figure 1) [3,16,17]. Importantly, cDC1s selectively express XCR1 and are the most efficient DCs in the cross-presentation of exogenous antigens to CD8^+^ T cells [22]. Of note, while cDC1s activate CCR7-expressing naïve CD8^+^ T cells [22], activated CD8^+^ T cells in turn produce XCL1, the ligand of XCR1 (Figure 1) [22]. This further promotes the interactions of CD8^+^ T cells and cDC1s, leading to full differentiation of effector CTLs [22]. In secondary immune responses, mature DCs also produce CCL3, CCL4, CCL5, CCL17, CCL22, CXCL9, CXCL10, and CXCL11 [22]. Since Th1 cells express CCR5 and CXCR3, while Th2 cells, Th17 cells, and Treg cells express CCR4 [22], these chemokine–chemokine receptor axes contribute to the rapid expansion of effector T cells (Figure 1).

## 3. Th1 Cell, CTL, and NK Cell

Th1 cells are involved in cellular immunity by secreting Th1-type cytokines such as interferon (IFN)-γ, Interleukin (IL)-2 and tumor necrosis factor (TNF)-α [23,24]. Th1-type cytokines promote the differentiation of naïve CD8^+^ T cells into CTLs [23,24] and also enhance the cytotoxic activity of CD8^+^ T cells and NK cells [25,26]. CD8^+^ T cells and NK cells are able to directly eliminate tumor cells by secreting cytotoxic molecules such as perforin and granzyme. NK cells are also able to induce apoptosis in tumor cells via TNF family molecules such as FAS ligand (FASL) and TNF-related apoptosis-inducing ligand (TRAIL) [27]. Thus, Th1 cells, CD8^+^ T cells, and NK cells are the major effector cells in anti-tumor immunity. Indeed, infiltration of CD8^+^ T cells and NK cells correlates with better clinical outcomes and therapeutic responses in various types of cancer [28,29]. In addition, recent studies have revealed the existence of CD8^+^ T cell subsets such as IFN-γ-expressing Tc1 cells, IL-4-expressing Tc2 cells, IL-9-expressing Tc9 cells, IL-17-expressing Tc17 cells, and IL-22-expressing Tc22 cells [30,31,32,33,34]. Tc1, Tc2, and Tc22 cells produce high levels of perforin and granzyme and have high cytotoxic activity, while Tc9 and Tc17 produce low levels of cytotoxic molecules and have poor cytotoxic activity [35,36]. The respective roles of these various Tc subsets in tumor immunity remain to be seen.

Th1 cells, CD8^+^ T cells, and NK cells commonly express CXCR3 [37,38,39]. Accordingly, these effector cells are recruited into tumor tissues by the CXCR3 ligands, CXCL9, CXCL10, and CXCL11 [25,26]. Previous studies have shown that intra-tumoral injection of CXCL9, CXCL10, CXCL11, or their fusion proteins efficiently induces the recruitment of CD8^+^ T cells and NK cells via CXCR3 and suppresses tumor growth in various murine cancer models such as lung cancer, colon cancer, kidney cancer, melanoma, and glioma [40,41]. It has also been reported that the expression of CXCL9, CXCL10, and CXCL11 is upregulated in the tumor tissues of patients with colon cancer, esophageal cancer, lung cancer, and ovarian cancer, and correlates with better clinical outcomes (Table 1) [42,43,44,45]. While CXCL9, CXCL10, and CXCL11 are weakly expressed by many types of cells including epithelial cells, endothelial cells, fibroblasts, and monocytes under physiological conditions, their expressions are strongly upregulated by IFN-γ and TNF-α [46,47,48]. CXCL9 is also known to be secreted by cDC1s and promotes the interactions of cDC1s with CXCR3-expressing CD8^+^ T cells, resulting in cluster formation and expansion of CD8^+^ T cells in the lymph nodes [11,49]. It has been further reported that T cell immunoglobulin and mucin domain containing-3 (TIM-3), a negative regulator of T cell responses, is highly expressed by myeloid cells including cDC1s in the tumor microenvironment, and treatment with anti-TIM-3 monoclonal antibody upregulates the production of CXCL9 by cDC1s, resulting in CD8^+^ T cell expansion and suppression of tumor growth in a murine breast cancer model [50]. Th1 cells, CD8^+^ T cells, and NK cells are also the major sources of IFN-γ, which induces the production of CXCL9 and CXCL10 by DCs and other tissue cells in the tumor microenvironment. This further attracts CXCR3-expressing Th1 cells, CD8^+^ T cells, and NK cells into tumor tissues by a positive feedback mechanism. Collectively, the CXCR3 axis plays vital roles in anti-tumor immunity through expansion and recruitment of Th1 cells, CD8^+^ T cells, and NK cells.

Th1 cells, CD8^+^ T cells, and NK cells also express CCR5 [37,38,39]. Its ligands CCL3, CCL4, and CCL5 are broadly expressed by a variety of normal cells and tumor cells, and frequently upregulated in tumor tissues of various types of cancer [55,56]. In particular, the increased expression of CCL5 has been shown to correlate with better clinical outcomes in several cancers including thyroid cancer, lung cancer, ovarian cancer, and melanoma (Table 1) [55,56]. Consistently, CCL5-overexpressing murine ovarian cancer and hepatocellular carcinoma showed reduced tumor growth by recruiting CCR5-expressing CD8^+^ T cells [57,58]. However, the increased expression of CCL5 has also been shown to correlate with poor clinical outcomes in breast cancer, pancreatic cancer, renal cancer, and glioma (Table 1) [57,58]. Accordingly, CCL5 is now considered as a marker for poor prognosis for breast cancer [59,60]. In this context, CCR5 is also expressed by immunosuppressive cells such as MDSCs and TAMs [52,79]. Indeed, CCL5-deficiency or CCR5 antagonist treatment suppressed tumor growth by reducing the recruitment of immunosuppressive MDSCs and TAMs into the tumor microenvironment in murine breast cancer models [80,81]. Thus, the CCL5-CCR5 axis may have both anti-tumor and pro-tumor activities depending on the types of responding cells.

CX3CR1 is also known to be selectively expressed by cytotoxic effector cells such as CD8^+^ T cells and NK cells [82,83]. Its ligand CX3CL1 is mainly expressed by epithelial cells, endothelial cells, DCs, and neurons, and upregulated by IFN-γ, TNF-α, and IL-1β [82,83,84]. CX3CL1 is a membrane-bound molecule and its soluble chemotactic form is produced by the cleavage in the membrane proximal region [46,85]. The production of soluble CX3CL1 by metalloproteinases such as ADAM10, ADAM17, and MMP-2 is enhanced under inflammatory conditions including cancer [84,86,87,88]. It has been reported that the increased expression of CX3CL1 correlates with the infiltration of CD8^+^ T cells and NK cells and better clinical outcomes in colon cancer, breast cancer, and lung cancer (Table 1) [71,72,73]. However, CX3CL1 has also been shown to promote angiogenesis through the recruitment of CX3CR1-expressing macrophages that secrete angiogenic factors in murine breast cancer, hepatocellular carcinoma, lung cancer, and melanoma models [74,75]. The expression levels of CX3CL1 have been shown to correlate with vascular density in the bone marrow of multiple myeloma patients [74]. Furthermore, CX3CL1 has been demonstrated to directly promote cell growth in gastric and pancreatic cancer cell lines via CX3CR1, although the detailed mechanism is unknown [89,90]. Thus, the CX3CL1-CX3CR1 axis may also have both anti-tumor and pro-tumor activities depending on the types of responding cells.

In addition, it has recently been reported that activated CD8^+^ T cells and NK cells secrete XCL1 and attract XCR1-expressing cDC1s to the tumor microenvironment [76,77]. Recruited cDC1s further activate CD8^+^ T cells and NK cells by producing IL-12 [76,77,78]. Currently, the XCL1-XCR1 axis has been attracting much attention for its role in induction and activation of antigen-specific CTLs and activation of NK cells [22].

## 4. Th2 Cell

Th2 cells are involved in humoral immunity by secreting Th2-type cytokines, such as IL-4, IL-5, IL-10, and IL-13 [91,92]. Th2 cells are shown to express CCR4, CCR8, and, to a lesser extent, CCR3 [46,85,93]. It has been reported that the expression levels of respective chemokine ligands are upregulated in some types of cancer, and Th2 cells are increased in tumor tissues and the draining lymph nodes [94]. Th2 cytokines inhibit the differentiation and function of Th1 cells, while Th1 cytokines inhibit the differentiation and function of Th2 cells [95]. Thus, Th2 cells may indirectly suppress anti-tumor immunity by negatively regulating Th1 responses. In particular, IL-10 is known to suppress Th1-mediated immune responses [96]. IL-4 has also been reported to enhance tumor growth by inhibiting apoptosis in a murine fibrosarcoma model [97]. On the other hand, it has also been demonstrated that adoptive transfer of tumor-specific Th2 cells protected mice against lethal challenge of murine myeloma and lymphoma through the induction of Th2-type inflammation in tumor tissues. However, Th2-mediated anti-tumor effects did not require B cells, NKT cells or CD8^+^ T cells and the mechanism was unknown [98]. Of note, Th2 cells are also known to secrete IL-31 [99]. Recently, it has been reported that IL-31-overexpressing murine breast carcinoma shows reduced tumor growth by inhibiting the activity of immunosuppressive cells such as Treg cells, MDSCs, and M2-type macrophages [100]. Collectively, Th2 cells are generally considered to have pro-tumor effects but may also have some anti-tumor activity.

## 5. Th17 Cell

**Figure 2:**
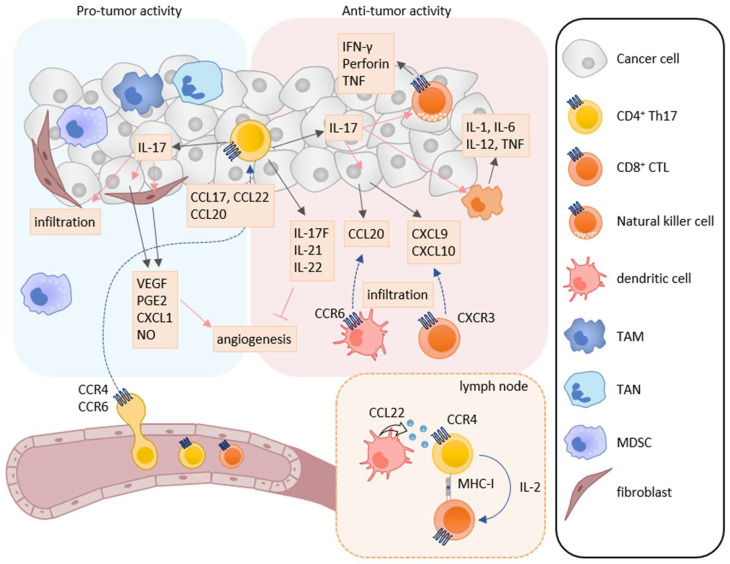
Roles of Th17 cells in tumor immunity. Th17 cells have both pro-tumor and anti-tumor activities in tumor immunity. IL-17A induces the production of angiogenic factors such as VEGF, PGE2, CXCL1, and NO from tumor cells and fibroblasts, leading to increase in angiogenesis. IL-17A also enhances the infiltration of MDSCs into tumor tissues. On the other hand, Th17 cells can directly induce tumor-specific CTLs via IL-2 production and MHC class I molecule expression in the lymph node. IL-17A also induces NK cells to express natural cytotoxicity receptor, perforin, TNF, and IFN-γ, and macrophages to express IL-1, IL-6, IL-12, and TNF. Furthermore, IL-17A induces the production of CXCL9 and CXCL10 in tumor cells and CCL20 in macrophages. These chemokines recruit CXCR3-expressing CTLs and CCR6-expressing immature DCs, respectively. In addition, IL-17F, IL-21, and IL-22 inhibit angiogenesis.

Th17 cells secrete the IL-17 family cytokines IL-17A, IL-17F, IL-21, IL-22, and granulocyte macrophage colony-stimulating factor (GM-CSF). Taken together, they play a critical role in immune responses against extracellular bacteria and fungi at mucosal tissues [101,102]. IL-17 is associated with the induction of pro-inflammatory immune responses by increasing the expression of other pro-inflammatory cytokines, chemokines, and chemical mediators in various types of cells [103,104]. Accordingly, Th17 cells have been shown to be involved in chronic inflammatory diseases such as psoriasis, rheumatoid arthritis, and multiple sclerosis [105,106]. Th17 cells have also been reported to infiltrate into tumor tissues of various cancers, and the infiltration of Th17 cells is associated with both better and poor clinical outcomes [107,108]. Thus, Th17 cells are now considered to have both pro-tumor and anti-tumor activities (Figure 2) [109].

### 5.1. Pro-Tumor Activity

A number of studies have shown the involvement of Th17 cells in tumor progression using several murine cancer models [110,111,112]. In humans, the infiltration of Th17 cells into tumor tissues correlates with poor clinical outcomes in cancers such as colon cancer, gastric carcinoma, hepatocellular carcinoma, non-small cell lung cancer, and pancreatic cancer [113,114,115,116,117]. While IL-17A-overexpressing murine fibrosarcoma and colon adenocarcinoma showed enhanced tumor growth with increased vascular density, IL-17A was shown to induce the production of various angiogenic factors including vascular endothelial growth factor (VEGF), prostaglandin E2, CXCL1, and nitric oxide by tumor cells and fibroblasts [118,119]. Consistently, it has been reported that the expression of IL-17A correlates with vascular density in the tumor tissues of various cancers such as colon cancer, gastric carcinoma, hepatocellular carcinoma, lung cancer, pancreatic cancer, and breast cancer [115]. IL-17A has also been shown to enhance the infiltration of MDSCs into tumor tissues and activate their immunosuppressive activity [120,121]. IL-17 has also been found to directly activate oncogenic pathways such as mitogen-activated protein kinase, nuclear factor-κB (NF-κB), and activator protein-1 (AP-1) signals, and to promote cell growth in gastric cancer and glioblastoma cell lines via IL-17 receptor [122,123]. In addition, Th17 cells express the cell surface ectonucleotidases CD39 and CD73 that generate adenosine, a well-known immunosuppressive mediator, from extracellular ATP derived from dying cells [124]. Adenosine inhibits proliferation and function of effector T cells via the adenosine A2A receptor [125].

Th17 cells express CCR4 and CCR6, which are considered to be involved in their recruitment into the tumor microenvironment [46,93,126,127]. Indeed, it has been reported that the expression levels of CCL17 and CCL22 are elevated in patients with esophageal squamous cell carcinoma and malignant ascites with increased infiltration of CCR4-expressing Th17 cells [128,129]. In addition, the expression levels of CCL20 have also been reported to be frequently elevated in tumor tissues of various types of cancer and to correlate with Th17 cell infiltration [127].

### 5.2. Anti-Tumor Activity

Although tumor infiltration of Th17 cells has been shown to correlate with poor clinical outcomes in some cancers [113,114,115,116,117], their infiltration has also been described to correlate with better overall survival in various cancers such as breast cancer, cervical carcinoma, esophageal squamous cell carcinoma, lung cancer, nasopharyngeal carcinoma, ovarian cancer, prostate cancer, renal cell carcinoma, and small cell lung cancer [130,131,132,133,134,135,136,137,138]. Conversely, reduced Th17 cells in ascitic fluid have been reported to correlate with poor clinical outcomes in ovarian cancer [139]. Furthermore, IL-17A-deficient mice exhibited enhanced tumor growth and lung metastasis in murine colon cancer and melanoma models [140,141]. Recent studies have further shown that Th17 cells are capable of directly inducing tumor-specific CTLs via IL-2 production and MHC class I expression in a murine melanoma model [141,142]. In adoptive transfer experiments, Th17 cells induced tumor-specific CTLs and suppressed tumor growth more efficiently than Th1 cells in a murine melanoma model [140,141]. Recent studies have also shown that the anti-tumor effect of Th17 cells requires IFN-γ-expressing cytotoxic CD8^+^ T cells in murine colon cancer, myeloma, and mastocytoma models [140,143]. Thus, Th17 cells may play an important role in the induction of tumor-specific CTL responses in certain types of cancer.

It has been reported that IL-17 enhances the expression of natural cytotoxicity receptors, perforin, TNF, and IFN-γ in NK cells and enhances the expression of IL-1, IL-6, IL-12, and TNF in macrophages [144,145,146]. In patients with ovarian cancer, IL-17 derived from Th17 cells has been shown to induce the production of CXCL9 and CXCL10 by tumor cells and tumor infiltrated macrophages, which recruits CXCR3-expressing CTLs and NK cells into tumor tissues [139]. Furthermore, Th17 cells have been shown to induce the production of CCL20 in tumor tissues, which recruits CCR6-expressing immature DCs to tumor tissues in a murine melanoma model. Consequently, tumor antigen-captured DCs migrate to draining lymph nodes and induce tumor-specific T cell responses [141]. While IL-17A promotes angiogenesis by increasing the production of VEGF, other Th17 cell derived cytokines such as IL-17F, IL-21, and IL-22 have been shown to have anti-angiogenic activities [147,148,149]. It has also been reported that IL-17 activates the caspase-dependent apoptosis signal in a breast cancer cell line [150].

As described above, Th17 cells predominantly express CCR4 and CCR6. Previously, we have shown that DCs in regional lymph nodes produce CCL22 and attract CCR4-expressing Th17 to enhance DC-Th17 interactions [46,85]. This promotes the expansion of Th17 cells and subsequent induction of CTLs in murine melanoma and psoriasis models [13,14]. Thus, in certain types of cancer, CCR4 may play a role in anti-tumor immunity not only by recruiting Th17 cells into tumor tissues but also by promoting Th17 cell expansion in the draining lymph nodes with subsequent Th17-mediated CTL induction. On the other hand, although CCR6 is a major trafficking receptor for Th17 cells and Treg cells [46,85], CCR6-deficient mice did not show any abnormalities in Th17 and Treg cell expansions in the same melanoma model [13]. Thus, CCR6 may be primarily involved in the recruitment of Th17 cells and Treg cells into tumor tissues.

## 6. Treg Cell

**Figure 3:**
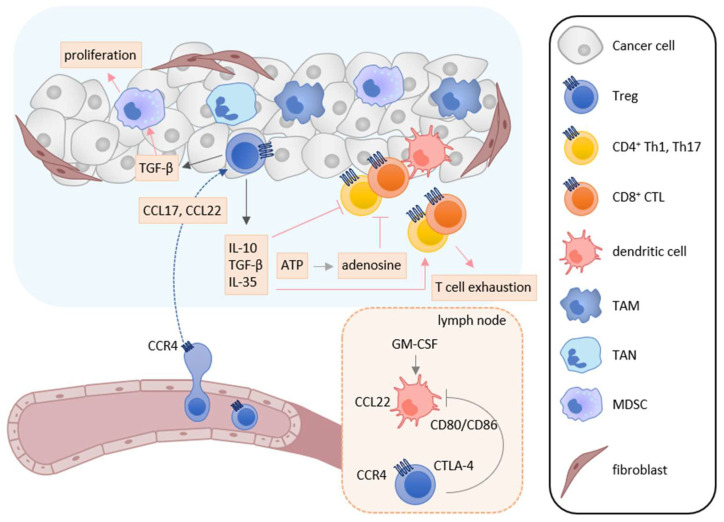
Roles of Treg cells in tumor immunity. In the tumor microenvironment, TAMs, TANs, tumor cells, and cancer-associated fibroblasts secrete CCL17 and/or CCL22, and recruit CCR4-expressing Treg cells into tumor tissues. Infiltrated Treg cells secret immunosuppressive cytokines such as IL-10, TGF-β, and IL-35. These cytokines suppress the activation and induction of tumor-specific T cell responses and induce T cell exhaustion. TGF-β also enhances the differentiation and immunosuppressive function of MDSCs. Furthermore, adenosine is generated from ATP derived from apoptotic Treg cells and inhibits the function and proliferation of effector T cells. Treg cells also inhibit the costimulatory signals of DCs to induce tumor-specific T cell responses by the CTLA-4-CD80/CD86 interaction.

Treg cells suppress immune responses under physiological and pathological conditions [151,152,153]. Numerous studies have shown that Treg cells inhibit the activation and proliferation of effector T cells and DCs, and play the major role in tumor escape from host immunosurveillance [151,152,153]. In the tumor microenvironment, infiltrated Treg cells secrete several immunosuppressive cytokines such as IL-10, TGF-β, and IL-35, which suppress the induction and activation of tumor-specific effector T cells (Figure 3) [152,153]. Furthermore, IL-10 and IL-35 derived from Treg cells promote the exhaustion of CD8^+^ tumor-infiltrating lymphocytes [154]. Treg cells also express cytotoxic T-lymphocyte antigen-4 (CTLA-4), a T cell inhibitory molecule, on their cell surface. CTLA-4 binds to costimulatory molecules CD80 and CD86 on DCs and thus blocks their binding to CD28 on T cells, resulting in inhibition of the costimulatory signals necessary for the induction of tumor-specific T cell responses [155]. Treg cells also express lymphocyte activation gene 3 (LAG-3), which inhibits the induction of tumor-specific T cell responses by suppressing DC activation via interaction with MHC class II [156,157]. Recently, cell metabolism has also been reported to be involved in the immunosuppressive mechanisms of Treg cells. While apoptotic Treg cells are a major source of ATP in the tumor microenvironment [158], Treg cells also express CD39 and CD73 and thus generate a large amount of adenosine from ATP in the tumor microenvironment, which inhibits the function and proliferation of effector T cells via the adenosine A2A receptor [125]. Treg cells also enhance the differentiation and immunosuppressive functions of MDSCs via the production of TGF-β [159]. In turn, MDSCs enhance the proliferation of Treg cells in the draining lymph nodes via the production of TGF-β [160].

Treg cells are known to express various chemokine receptors, including CCR4, CCR5, CCR6, CCR8, CCR10, and CXCR3 [46,85,93,161]. Among them, CCR4 is the most well characterized trafficking receptor for Treg recruitment to the tumor microenvironment [3,61,161]. Many studies have shown that CCL17 and CCL22 are highly expressed in various tumor tissues including lung cancer, colorectal cancer, gastric cancer, breast cancer, and ovarian cancer [61,162,163]. In the tumor tissues, CCL17 and CCL22 are mainly secreted by macrophages and tumor cells [164,165,166,167,168]. CCL17 is also secreted by immunosuppressive tumor-associated neutrophils (TANs) and cancer-associated fibroblasts [169,170,171]. Consistently, it has been reported that the increased expression of CCL17 and CCL22 correlates with the infiltration of CCR4-expressing Treg cells and poor clinical outcomes in gastric cancer, breast cancer, and oral tongue squamous cell carcinoma (Table 1) [62,162,172]. In this regard, mogamulizumab, an anti-CCR4 monoclonal antibody, was recently developed for the treatment of CCR4-expressing adult T-cell leukemia/lymphoma [93]. The mogamulizumab treatment was also shown to efficiently deplete CCR4-expressing Treg cells and to increase the number of tumor-specific CD8^+^ T cells in the blood of patients with adult T-cell leukemia/lymphoma [173,174]. It was further shown that a CCR4 antagonist CCR4-351 inhibited Treg cell recruitment into tumor tissues in mice bearing murine pancreatic and colon cancers, and augmented tumor-specific immune responses [63]. In addition, the combination treatment of another CCR4 antagonist piperidinyl-azetidines and immune checkpoint inhibitors, such as anti-PD-L1 and CTLA-4 antibodies, efficiently augmented tumor-specific immune responses [175]. We have also shown that co-injection of compound 22, a CCR4 antagonist, with cancer vaccines inhibits Treg cell recruitment into vaccination sites and Treg-mediated DC suppression, resulting in enhanced induction of tumor-specific CTLs in a murine melanoma model [176].

Antigen-captured DCs are known to abundantly secrete CCL22 and CCL17. Furthermore, GM-CSF derived from T cells is a potent inducer of CCL22 expression by DCs in the lymph nodes [177]. Accordingly, the CCR4 axis plays an important role in the expansion of CCR4-expressing T cells by mediating their interactions with DCs [46,178]. Indeed, it has been reported that CCL22 secreted from DCs promotes the interactions of DCs and CCR4-expressing Treg cells, leading to the expansion of Treg cells in the lymph nodes [12]. Thus, the CCR4 axis may be involved in Treg cell expansion in the draining lymph nodes and Treg-mediated DC suppression in the tumor microenvironment.

CCR6 is broadly expressed on many types of immune cells, including immature DCs, B cells, effector/memory T cells, NK cells, and NKT cells [46,85]. CCR6 is also highly expressed by a fraction of Treg cells [179]. Its ligand CCL20 is broadly produced by epithelial cells, endothelial cells, and several immune cells such as Th17 cells, B cells, NK cells, and neutrophils [127,180,181]. The expression of CCL20 has been reported to be increased in tumor tissues of many types of cancer including breast cancer, colon cancer, skin cancer, oral cancer, and prostate cancer [127,182]. CCL20 is also frequently expressed by various types of cancer including hepatocellular carcinoma, breast cancer, colon cancer, pancreatic cancer, prostate cancer, lung cancer, and renal cell carcinoma [127,180,181]. Furthermore, the infiltration of CCR6-expressing Treg cells is confirmed to correlate with poor clinical outcomes in colorectal cancer, non-small cell lung cancer, oral squamous cell carcinoma, and esophageal squamous cell carcinoma (Table 1) [64,65,66,67]. Thus, the CCL20-CCR6 axis may have a pro-tumor activity by recruiting Treg cells to the tumor microenvironment.

CCR8 is expressed by a fraction of Treg cells, and its ligand CCL1 is capable of inducing efficient migration of CCR8-expressing Treg cells [183,184]. It was also reported that CCL1 was produced by Treg cells in a murine experimental autoimmune encephalomyelitis model [185]. Of note, CCL1 was shown to induce the expression of CCR8, Foxp3, CD39, granzyme B, and IL-10 in Treg cells [185]. Furthermore, the infiltration of CCR8-expressing Treg cells in the tumor microenvironment correlates with poor clinical outcomes in breast cancer (Table 1) [51]. Thus, the CCL1-CCR8 axis may recruit Treg cells and also maintain Treg cell phenotype and function.

In addition, it has been reported that hypoxia induces the expression of CCL28, a CCR10 ligand, by tumor cells of ovarian and liver cancers, which recruits CCR10-expressing Treg cells into tumor tissues [68,69]. Furthermore, CCR10-expressing Treg cells secrete VEGF and contribute to angiogenesis. In consistence with these findings, the infiltration of CCR10-expressing Treg cells correlates with poor clinical outcomes in these cancers (Table 1) [68,69]. Thus, the CCL28-CCR10 axis may have significant pro-tumor activities in the hypoxic tumor microenvironment by inducing angiogenesis via VEGF and attracting CCR10-expressing Treg cells.

## 7. TAM, MDSC, and TAN

TAMs are known to be abundantly present in the tumor microenvironment [6,7]. Many studies have shown that TAMs have a multiple role in tumor progression. TAMs enhance immunosuppression, tumor cell growth, metastasis, and angiogenesis by inducing the expression of cytokines, chemokines, and growth factors [186,187]. Consistently, TAM infiltration into the tumor microenvironment correlates with poor prognosis in most solid cancers [188,189]. Although TAMs express various chemokine receptors, the CCL2-CCR2 axis plays a dominant role in their recruitment into tumor tissues [10,52]. In addition, TAMs also utilize CCR5 and CXCR4 for their recruitment in some types of cancer [10,52]. MDSCs are also abundantly present in the tumor microenvironment [6,7]. MDSCs are a heterogenous population of immune cells and can be broadly divided into two categories: monocytic-MDSCs and polymorphonuclear-MDSCs [6,7]. Both MDSCs contribute to tumor progression by enhancing immunosuppression, angiogenesis, and epithelial–mesenchymal transition [6,7]. Monocytic-MDSCs indirectly and nonspecifically inhibit the activity of many types of effector cells by producing immunosuppressive mediators such as reactive nitrogen species, inducible nitric oxide synthase and arginase [190,191]. Furthermore, infiltrated monocytic-MDSCs have been shown to be able to differentiate into TAMs in the tumor microenvironment [190,191]. On the other hand, polymorphonuclear-MDSCs directly interact with CD8^+^ T cells and inhibit antigen-specific CD8^+^ T cell responses by producing reactive oxygen species in the draining lymph nodes [190,191]. Monocytic-MDSCs and polymorphonuclear-MDSCs utilize similar chemokine receptors as monocytes and neutrophils, respectively. Monocytic-MDSCs express CCR2 and CCR1/CCR5, and can be recruited into tumor tissues by CC chemokines such as CCL2 and CCL5, respectively [79,192]. On the other hand, polymorphonuclear-MDSCs express CXCR2 and can be recruited into tumor tissues by CXC chemokines such as CXCL8 [70,190,193]. MDSCs also express CXCR4 and can be recruited into tumor tissues by CXCL12 [70,190,193]. TANs have the same developmental origin and similar phenotypical features as polymorphonuclear-MDSCs [194,195]. TANs have pro-tumor activity by producing immunosuppressive soluble mediators such as TGF-β and arginase [194,195]. Similar to normal neutrophils, TANs utilize the CXCR2 axis for their recruitment [196]. In addition, TAMs, monocytic-MDSCs, polymorphonuclear-MDSCs, and TANs can produce the CCR4 ligands CCL17 and CCL22 in tumor tissues, contributing to tumor progression by recruiting CCR4-expressing Treg cells [3].

## 8. The Chemokine Superfamily as Therapeutic Targets in Cancer Immunotherapy

Although the chemokine superfamily have long been regarded as highly promising therapeutic targets for drug development, currently only three drugs are approved: Maraviroc, a small-molecule CCR5 antagonist for blocking infection by CCR5-tropic HIV-1; Plerixafor, a small-molecule CXCR4 antagonist for the mobilization of hematopoietic stem cells from the bone marrow for transplantation in patients with non-Hodgkin’s lymphoma and multiple myeloma; Mogamulizumab, a fully humanized and glyco-engineered monoclonal anti-CCR4 antibody for patients with aggressive/refractory adult T cell leukemia/lymphoma (ATLL) and cutaneous T cell lymphomas (CTCLs) [93,173,197]. However, the recent impressive therapeutic success of immune checkpoint inhibitors in cancer immunotherapy have opened the possibility of clinical application of drugs targeting chemokines and chemokine receptors as an adjunct therapeutic drug for cancer immunotherapy or chemotherapy. Indeed, as summarized in a recent review, a number of preclinical studies have shown significant therapeutic effects of drugs targeting chemokine receptors in cancer immunotherapy of various murine cancer models, and some of these approaches are currently being tested in clinical trials [198,199]. Here, we highlight the recent development in preclinical studies (Table 2) and clinical studies (Table 3) involving the chemokine superfamily.

### 8.1. CXCR3

CXCR3 plays a major role in tissue recruitment of Th1 cells, CTLs, and NK cells. Accordingly, enhanced production of the CXCR3 ligands in tumor tissues is considered to be beneficial in cancer patients [25,26]. As described in Section 3, CXCL9 production by cDC1s is negatively regulated by TIM-3 on their cell surface, and clinical trials are currently being conducted for several anti-TIM-3 monoclonal antibodies such as cobolimab, MBG453, LY3321367, and BMS986258, aiming at the upregulation of CXCL9 expression in the tumor microenvironment [217]. Some clinically available anticancer drugs including ipilimumab (an anti-CTLA-4), doxorubicin, and dacarbazine have also been shown to upregulate the expression levels of CXCL9, CXCL10, and CXCL11 in the tumor tissues of patients with melanoma and breast cancer, possibly contributing to their therapeutic effects [218,219,220].

### 8.2. CCR4 and CCR8

CCR4 is the major trafficking receptors for Treg cells and has been implicated in their recruitment into the tumor microenvironment [164,165,166,167,168]. In this context, several CCR4-targeted cancer immunotherapies have been performed in both animal and human studies, aiming at inhibiting Treg cell-mediated immune suppression [174,202,221]. In particular, mogamulizumab, an anti-CCR4 monoclonal antibody with a potent antibody-dependent cellular cytotoxicity (ADCC) activity, has been developed and approved for the treatment of CCR4-expressing T cell malignancies such as ATLL and CTCLs with great success [173]. Of note, mogamulizumab treatment has also shown to efficiently deplete Treg cells in patients with ATLL and CTCLs [173] and to increase tumor-specific CD8^+^ T cells in the blood of patients with ATLL [174,202]. Thus, blocking CCR4 by mogamulizumab has been regarded as a promising strategy to enhance tumor-specific immune responses by depleting Treg cells. Accordingly, several clinical trials have been performed to test the efficacy of combination of mogamulizumab (KW-0761) and one of the immune checkpoint inhibitors in patients with solid tumors. Although it has been confirmed that Treg cells in the peripheral blood and tumor tissues are efficiently depleted in patients treated with mogamulizumab, no synergistic enhancement of the therapeutic effect of immune checkpoint inhibitors by the combination with mogamulizumab has been observed so far [173]. Importantly, CCR4 is also expressed by other T cell subsets including Th17 cells. While initial studies have suggested protumor activities of Th17 cells, recent studies have demonstrated an important role of Th17 in the induction of CTLs [140,141,142,143]. Our previous studies have further shown that CCR4 plays an essential role in Th17 expansion and subsequent CTL induction by promoting DC-Th17 interactions in the regional lymph nodes [13,14]. Thus, CCR4 may also substantially contribute to anti-tumor immunity through the expansion of Th17 cells and subsequent induction of tumor-specific CTLs in certain types of cancer. Accordingly, CCR4-targeted cancer immunotherapy aiming at Treg depletion may also suppress anti-tumor immunity by depleting other T cell subsets including Th17 cells. Furthermore, patients treated with mogamulizumab often experience severe adverse events such as skin rashes most probably due to subclinical autoimmune responses unleashed by Treg depletion and also a profound lymphopenia, often resulting in opportunistic infections [173]. Thus, although the highly enhanced ADCC activity of mogamulizumab is valuable for the efficient elimination of CCR4-expressing malignant T cells, such a potent ADCC activity may not be necessary for the functional depletion of Treg cells in patients with solid tumors [173]. Thus, it remains to be seen whether other anti-CCR4 monoclonal antibodies or small molecular CCR4 antagonists may have better effects on Treg suppression in solid tumors [222].

CCR8 is also expressed by Treg cells and its expression is relatively selective for Treg cells and minimal in other immune cells [183,184]. CCR8-expressing Treg cells are also considered to have augmented suppressor activity [185,223]. Indeed, CCR8 has been shown to play a pivotal role in tumor progression by regulating the localization and function of Treg cells in murine cancer models [185]. Recent preclinical studies have further demonstrated that anti-CCR8 antibodies efficiently deplete Treg cells and suppress tumor growth in murine colon cancer and bladder carcinoma models [40,41]. Accordingly, several anti-CCR8 humanized monoclonal antibodies, such as SRF114, HBM1022, and FPA157, have been developed and shown to deplete Treg cells by ADCC [183,184]. Thus, the treatment with anti-CCR8 may provide a new option for Treg depletion in cancer immunotherapy without major side effects.

### 8.3. CXCR2 and CXCR4

CXCR2 has been shown to play a major role in tumor progression by recruiting polymorphonuclear type MDSCs and TANs into the tumor microenvironment in murine models of cancers such as colon cancer, pancreatic cancer, head and neck cancer, and melanoma [196,224,225,226]. A recent study has shown that inhibition of CXCR2 using SB225002, a CXCR2 inhibitor, significantly decreases infiltration of TANs in tumor tissues and suppresses tumor growth in a murine lung cancer model [196]. However, CXCR2 is a major trafficking receptor for neutrophils and thus blocking CXCR2 may increase susceptibility to bacterial infection. Regarding this, however, it has been reported that in humans, AZD5069, another CXCR2 inhibitor, shows little adverse effect on the mobilization of neutrophils from the bone marrow or on their phagocytosis and oxidative burst in response to bacterial pathogens [227]. Thus, blocking infiltration of polymorphonuclear-MDSCs and TANs in the tumor microenvironment by CXCR2 inhibitors may provide a safe and promising treatment option in cancer immunotherapy.

CXCR4 is another major trafficking receptor for MDSCs and several clinical trials of CXCR4 inhibitors have been conducted for the treatment of some cancers [213,214,215,216]. In particular, BL-8040, a CXCR4 inhibitor, in combination with anti-PD-1 monoclonal antibody or chemotherapy has been shown to efficiently decrease MDSCs and increase CD8^+^ effector T cells in tumor tissues in patients with pancreatic cancer, resulting in better clinical outcomes [214]. Although CXCR4 is widely expressed not only by immune cells but also various tissue cells including tumor cells and stromal cells [46,85], recent clinical studies have shown that the CXCR4 inhibitors including BL-8040, balixafortide, and LY2510924 are safe and tolerable in humans [214,215,216]. Thus, CXCR4 inhibitors may also provide a promising approach for suppression of MDSCs in cancer immunotherapy.

### 8.4. CCR2 and CCR5

CCR2 is a major traffic receptor for TAMs. CCR2 blocking has been shown to suppress tumor growth in murine liver cancer and pancreatic ductal adenocarcinoma models [228]. However, CCR2 is also known to be widely expressed by immune cells and in particular by monocytes and macrophages [229]. Furthermore, a recent clinical study of PF-04136309, a CCR2 inhibitor, in combination with nab-paclitaxel and gemcitabine has found severe adverse effects by this regimen including pulmonary toxicity in patients with pancreatic ductal adenocarcinoma [209].

CCR5 is also expressed by TAMs and MDSCs in the tumor microenvironment [52,79]. Since CCR5 inhibitors are clinically used for prevention of HIV-1 infection, the re-tasking of CCR5 inhibitors for cancer immunotherapy may be an interesting approach [230]. However, since CCR5 is also expressed by Th1 cells, CTLs, and NK cells, CCR5 blockade may also suppress anti-tumor immunity mediated by these effector cells.

### 8.5. XCR1

cDC1s are now known to be the most efficient DC subset for the induction of CTLs not only to intracellular pathogens but also to extracellular antigens via cross-presentation [3,16,17,19]. Furthermore, cDC1s uniquely express XCR1, while its ligand XCL1 is mainly produced by activated CD8^+^ T cells and NK cells [22]. Thus, taking advantage of this highly selective expression of XCR1 on cDC1s and their well-known ability for cross-presentation, many research groups have tested target delivery of vaccine antigens by using XCL1-fusion proteins with substantial success [22]. On the other hand, we considered using XCL1 itself as an adjuvant to attract XCR1-expressing cDC1s into the injection site of cancer vaccines for efficient antigen delivery to cDC1s. To test this possibility, we first generated a stabilized form of XCL1 by introducing a second C-C bond, since the natural XCL1 is a weak chemoattractant because of its unstable structure due to the lack of the second and forth cysteine residues conserved in other chemokines [231]. We have shown that the stable form of XCL1 is highly chemotactic and efficiently induces antigen-specific CTL responses by attracting cDC1s when co-injected with cancer vaccines [232,233]. Thus, the XCL1-XCR1 axis may provide a new opportunity for efficient induction of anti-tumor CTLs by cancer vaccines [22].

## 9. Conclusions

As described in this article, a diverse array of immune cells is now known to be involved in host tumor immunity and infiltrates into tumor tissues, generating a highly complex cell population in the tumor microenvironment [198]. Immune cells can have either anti-tumor or pro-tumor activities depending on their effector functions. These cells also express selective but often overlapping chemokine receptors and infiltrate into tumor tissues via chemokines produced by cells including tumor cells, infiltrating immune cells, and stromal cells [198]. To make matters more complex, tumor cells often express several chemokine receptors, and the corresponding chemokines are potentially involved in tumor cell functions such as proliferation, metastasis, and stemness [10,234]. Furthermore, it is known that some chemokines are positively and others negatively involved in angiogenesis [235]. Thus, targeting chemokines or chemokine receptors for cancer immunotherapy is an attractive but highly challenging task. However, after the recent success of immune checkpoint inhibitors in cancer immunotherapy, drugs targeting chemokines or chemokine receptors may have a new possibility as an adjunct drug for the main cancer immunotherapy. This is partly because the immune checkpoint inhibitors are beneficial in only a fraction of patients and one of the mechanisms of such primary resistance is considered to be the presence of potent immunosuppressive effector cells such as Treg cells and MDSCs in the tumor microenvironment. Thus, drugs targeting chemokines or chemokine receptors may provide a new tool to functionally suppress immunosuppressive cells. Indeed, some recent preclinical (Table 2) and clinical studies (Table 3) with drugs blocking chemokine receptors, such as CCR4, CCR8, CXCR2, and CXCR4, have shown some promising results and warrant further studies [196,214]. Collectively, it is hoped that drugs targeting the members of the chemokine superfamily may have a successful clinical application in cancer immunotherapy in near future.

## Figures and Tables

**Table 1:** Expression of chemokines and their roles in the tumor microenvironment.

Chemokine	Receptor	Target Cell	Tumor Type	Function	Status	Ref
CCL1	CCR8	Treg	breast cancer	Immune suppression	Pro-tumor	[51]
CCL2	CCR2	MDSC TAM	pancreatic ductal adenocarcinoma, breast cancer, lung cancer, renal cell carcinoma, endometrial cancer	Immune suppression Angiogenesis	Pro-tumor	[52,53,54]
CCL5	CCR5	CD8^+^ T cell	thyroid cancer, lung cancer, ovarian cancer, melanoma	Cytotoxic activity	Anti-tumor	[55,56]
		MDSC TAM	breast cancer, pancreatic cancer, renal cancer, glioma	Immune suppression	Pro-tumor	[57,58,59,60]
CCL17 CCL22	CCR4	Treg	gastric cancer, breast cancer, oral tongue squamous carcinoma	Immune suppression	Pro-tumor	[61,62,63]
CCL20	CCR6	Treg	colorectal cancer, non-small cell lung cancer, oral squamous cell carcinoma, esophageal squamous cell carcinoma	Immune suppression	Pro-tumor	[64,65,66,67]
CCL28	CCR10	Treg	ovarian cancer, liver cancer	Angiogenesis	Pro-tumor	[68,69]
CXCL8	CXCR2	TAN	colorectal cancer	Immune suppression	Pro-tumor	[70]
CXCL9 CXCL10 CXCL11	CXCR3	Th1 cell CD8^+^ T cell NK cell	colon cancer, esophageal cancer, lung cancer, ovarian cancer	Cytotoxic activity	Anti-tumor	[42,43,44,45]
CXCL12	CXCR4	TAM	lung cancer, glioma	Immune suppression	Pro-tumor	[52]
CX3CL1	CX3CR1	CD8^+^ T cell NK cell	colon cancer, breast cancer, lung cancer	Cytotoxic activity	Anti-tumor	[71,72,73]
		Macrophage	breast cancer, hepatocellular carcinoma, lung cancer, melanoma	Angiogenesis	Pro-tumor	[74,75]
XCL1	XCR1	cDC1	melanoma, breast cancer, lung cancer, head and neck squamous cell carcinoma	CD8^+^ T induction	Anti-tumor	[76,77,78]

**Table 2:** Therapeutic effects of chemokine receptor inhibitors in preclinical studies.

Target	Inhibitor	Tumor Type	Outcome	Reference
CCR2	CCX872 + anti-PD-1	Murine glioma model	Decrease MDSC Enhance anti-PD-1 effect Antitumor effect	[200]
	RS504393 + anti-PD-1	Murine breast cancer and melanoma models	Decrease TAM Antitumor effect	[201]
CCR4	Mogamulizumab + Piroxicam	Canine bladder cancer model	Decrease Treg cell Antitumor effect	[202]
	CCR4-351 + anti-CTLA-4 or anti-4-1BB	Murine pancreatic cancer model	Decrease Treg cell Antitumor effect	[63]
CCR5	mCCR5–Ig fusion antibody	Murine melanoma model	Decrease MDSC Antitumor effect	[203]
CCR8	JTX-1811	Multiple murine cancer models	Deplete tumor Treg cell but not normal tissue Treg cell Antitumor effect	[204]
	SRF114	In vitro study	Deplete Treg cell	[205]
	HBM1022 alone or + Pembrolizumab	Multiple murine cancer models	Deplete Treg cell Antitumor effect	[206]
	FPA157	Murine colon cancer models	Deplete Treg cell Antitumor effect	[207]
	25B3	Multiple murine cancer models	Deplete Treg cell Antitumor effect	[208]
CXCR2	SB225002 + Cisplatin	Murine lung cancer model	Decrease neutrophil Enhance anti-tumor CD8^+^ T cell activation Antitumor effect	[196]

Mogamulizumab (anti-CCR4); Pembrolizumab (anti-PD-1).

**Table 3:** Therapeutic effects of chemokine receptor inhibitors in clinical trials.

Target	Inhibitor	Tumor type	Outcome	Reference
CCR2	PF-04136309 + nab-Paclitaxel + Gemcitabine	Untreated metastatic pancreatic ductal adenocarcinoma	Phase Ib/II Enhanced pulmonary toxicity No enhanced antitumor efficacy	[209], NCT02732938
CCR4	FLX475 + Pembrolizumab	Breast cancer	Phase I/II	NCT03674567
	Mogamulizumab + Utomilumab	Advanced solid tumors	Phase Ib Safe and tolerable	[210], NCT02444793
	Mogamulizumab + Durvalumab or Tremelimumab	Advanced solid tumors	Phase I Depletion of Treg cells No enhanced antitumor efficacy	[211], NCT02301130
	Mogamulizumab + Nivolumab	Advanced or metastatic solid tumors	Phase I Acceptable safety profile Depletion of Treg cells Increases in CD8^+^ T cells;Potentially effective	[212], NCT02476123
CXCR4	AMD3100	Microsatellite stable pancreatic or colorectal cancer	Phase I Immunosuppression	[213], NCT02179970
	BL-8040 + Pembrolizumab + Chemotherapy	Metastatic pancreatic ductal adenocarcinoma	Phase II Depletion of MDSCs and Treg cells Increases in effector CD8^+^ T cells Enhanced effect of chemotherapy	[214], NCT02826486
	Balixafortide + Eribulin	Metastatic breast cancer	Phase I Safe and tolerable	[215], NCT01837095
	LY2510924 + Durvalumab	Advanced refractory solid tumor	Phase Ia Safe and tolerable	[216], NCT02737072

Pembrolizumab (anti-PD-1); Mogamulizumab (anti-CCR4); Utomilumab (anti-4-1BB); Durvalumab (anti-PD-L1); Tremelimumab (anti-CTLA-4); Nivolumab (anti-PD-1).

## References

[1] Chen D.S., Mellman I. (2013). Oncology meets immunology: The cancer-immunity cycle. Immunity.

[2] Lin J.X., Leonard W.J. (2019). Fine-Tuning Cytokine Signals. Annu. Rev. Immunol..

[3] Ozga A.J., Chow M.T., Luster A.D. (2021). Chemokines and the immune response to cancer. Immunity.

[4] Zhu X., Zhu J. (2020). CD4 T Helper Cell Subsets and Related Human Immunological Disorders. Int. J. Mol. Sci..

[5] Chatzileontiadou D.S.M., Sloane H., Nguyen A.T., Gras S., Grant E.J. (2020). The Many Faces of CD4(+) T Cells: Immunological and Structural Characteristics. Int. J. Mol. Sci..

[6] Szebeni G.J., Vizler C., Nagy L.I., Kitajka K., Puskas L.G. (2016). Pro-Tumoral Inflammatory Myeloid Cells as Emerging Therapeutic Targets. Int. J. Mol. Sci..

[7] Okla K., Wertel I., Polak G., Surowka J., Wawruszak A., Kotarski J. (2016). Tumor-Associated Macrophages and Myeloid-Derived Suppressor Cells as Immunosuppressive Mechanism in Ovarian Cancer Patients: Progress and Challenges. Int. Rev. Immunol..

[8] Quail D.F., Joyce J.A. (2013). Microenvironmental regulation of tumor progression and metastasis. Nat. Med..

[9] Sarvaiya P.J., Guo D., Ulasov I., Gabikian P., Lesniak M.S. (2013). Chemokines in tumor progression and metastasis. Oncotarget.

[10] Do H.T.T., Lee C.H., Cho J. (2020). Chemokines and their Receptors: Multifaceted Roles in Cancer Progression and Potential Value as Cancer Prognostic Markers. Cancers (Basel).

[11] Kastenmuller W., Brandes M., Wang Z., Herz J., Egen J.G., Germain R.N. (2013). Peripheral prepositioning and local CXCL9 chemokine-mediated guidance orchestrate rapid memory CD8+ T cell responses in the lymph node. Immunity.

[12] Rapp M., Wintergerst M.W.M., Kunz W.G., Vetter V.K., Knott M.M.L., Lisowski D., Haubner S., Moder S., Thaler R., Eiber S. (2019). CCL22 controls immunity by promoting regulatory T cell communication with dendritic cells in lymph nodes. J. Exp. Med..

[13] Matsuo K., Itoh T., Koyama A., Imamura R., Kawai S., Nishiwaki K., Oiso N., Kawada A., Yoshie O., Nakayama T. (2016). CCR4 is critically involved in effective antitumor immunity in mice bearing intradermal B16 melanoma. Cancer Lett..

[14] Matsuo K., Kitahata K., Kaibori Y., Arima Y., Iwama A., Ito M., Hara Y., Nagakubo D., Quan Y.S., Kamiyama F. (2021). CCR4 Involvement in the Expansion of T Helper Type 17 Cells in a Mouse Model of Psoriasis. J. Investig. Dermatol..

[15] Schumann K., Lammermann T., Bruckner M., Legler D.F., Polleux J., Spatz J.P., Schuler G., Forster R., Lutz M.B., Sorokin L. (2010). Immobilized chemokine fields and soluble chemokine gradients cooperatively shape migration patterns of dendritic cells. Immunity.

[16] Katou F., Ohtani H., Nakayama T., Nagura H., Yoshie O., Motegi K. (2003). Differential expression of CCL19 by DC-Lamp+ mature dendritic cells in human lymph node versus chronically inflamed skin. J. Pathol..

[17] Johnson L.A., Jackson D.G. (2014). Control of dendritic cell trafficking in lymphatics by chemokines. Angiogenesis.

[18] Willimann K., Legler D.F., Loetscher M., Roos R.S., Delgado M.B., Clark-Lewis I., Baggiolini M., Moser B. (1998). The chemokine SLC is expressed in T cell areas of lymph nodes and mucosal lymphoid tissues and attracts activated T cells via CCR7. Eur. J. Immunol..

[19] Luther S.A., Tang H.L., Hyman P.L., Farr A.G., Cyster J.G. (2000). Coexpression of the chemokines ELC and SLC by T zone stromal cells and deletion of the ELC gene in the plt/plt mouse. Proc. Natl. Acad. Sci. USA.

[20] Baekkevold E.S., Yamanaka T., Palframan R.T., Carlsen H.S., Reinholt F.P., von Andrian U.H., Brandtzaeg P., Haraldsen G. (2001). The CCR7 ligand elc (CCL19) is transcytosed in high endothelial venules and mediates T cell recruitment. J. Exp. Med..

[21] Sallusto F., Lanzavecchia A. (2000). Understanding dendritic cell and T-lymphocyte traffic through the analysis of chemokine receptor expression. Immunol. Rev..

[22] Matsuo K., Yoshie O., Kitahata K., Kamei M., Hara Y., Nakayama T. (2021). Recent Progress in Dendritic Cell-Based Cancer Immunotherapy. Cancers (Basel).

[23] Mosmann T.R., Coffman R.L. (1989). TH1 and TH2 cells: Different patterns of lymphokine secretion lead to different functional properties. Annu. Rev. Immunol..

[24] Murphy K.M., Ouyang W., Farrar J.D., Yang J., Ranganath S., Asnagli H., Afkarian M., Murphy T.L. (2000). Signaling and transcription in T helper development. Annu. Rev. Immunol..

[25] Alspach E., Lussier D.M., Schreiber R.D. (2019). Interferon gamma and Its Important Roles in Promoting and Inhibiting Spontaneous and Therapeutic Cancer Immunity. Cold Spring Harb. Perspect. Biol..

[26] Mortara L., Balza E., Bruno A., Poggi A., Orecchia P., Carnemolla B. (2018). Anti-cancer Therapies Employing IL-2 Cytokine Tumor Targeting: Contribution of Innate, Adaptive and Immunosuppressive Cells in the Anti-tumor Efficacy. Front. Immunol..

[27] Wallin R.P., Screpanti V., Michaelsson J., Grandien A., Ljunggren H.G. (2003). Regulation of perforin-independent NK cell-mediated cytotoxicity. Eur. J. Immunol..

[28] Corgnac S., Boutet M., Kfoury M., Naltet C., Mami-Chouaib F. (2018). The Emerging Role of CD8(+) Tissue Resident Memory T (TRM) Cells in Antitumor Immunity: A Unique Functional Contribution of the CD103 Integrin. Front. Immunol..

[29] Nersesian S., Schwartz S.L., Grantham S.R., MacLean L.K., Lee S.N., Pugh-Toole M., Boudreau J.E. (2021). NK cell infiltration is associated with improved overall survival in solid cancers: A systematic review and meta-analysis. Transl. Oncol..

[30] St Paul M., Ohashi P.S. (2020). The Roles of CD8(+) T Cell Subsets in Antitumor Immunity. Trends Cell Biol..

[31] Kemp R.A., Ronchese F. (2001). Tumor-specific Tc1, but not Tc2, cells deliver protective antitumor immunity. J. Immunol..

[32] Visekruna A., Ritter J., Scholz T., Campos L., Guralnik A., Poncette L., Raifer H., Hagner S., Garn H., Staudt V. (2013). Tc9 cells, a new subset of CD8(+) T cells, support Th2-mediated airway inflammation. Eur. J. Immunol..

[33] Yen H.R., Harris T.J., Wada S., Grosso J.F., Getnet D., Goldberg M.V., Liang K.L., Bruno T.C., Pyle K.J., Chan S.L. (2009). Tc17 CD8 T cells: Functional plasticity and subset diversity. J. Immunol..

[34] St Paul M., Saibil S.D., Lien S.C., Han S., Sayad A., Mulder D.T., Garcia-Batres C.R., Elford A.R., Israni-Winger K., Robert-Tissot C. (2020). IL6 Induces an IL22(+) CD8(+) T-cell Subset with Potent Antitumor Function. Cancer Immunol. Res..

[35] Lu Y., Hong B., Li H., Zheng Y., Zhang M., Wang S., Qian J., Yi Q. (2014). Tumor-specific IL-9-producing CD8+ Tc9 cells are superior effector than type-I cytotoxic Tc1 cells for adoptive immunotherapy of cancers. Proc. Natl. Acad. Sci. USA.

[36] Huber M., Heink S., Grothe H., Guralnik A., Reinhard K., Elflein K., Hunig T., Mittrucker H.W., Brustle A., Kamradt T. (2009). A Th17-like developmental process leads to CD8(+) Tc17 cells with reduced cytotoxic activity. Eur. J. Immunol..

[37] Bonecchi R., Bianchi G., Bordignon P.P., D’Ambrosio D., Lang R., Borsatti A., Sozzani S., Allavena P., Gray P.A., Mantovani A. (1998). Differential expression of chemokine receptors and chemotactic responsiveness of type 1 T helper cells (Th1s) and Th2s. J. Exp. Med..

[38] Rabin R.L., Park M.K., Liao F., Swofford R., Stephany D., Farber J.M. (1999). Chemokine receptor responses on T cells are achieved through regulation of both receptor expression and signaling. J. Immunol..

[39] Campbell J.J., Qin S., Unutmaz D., Soler D., Murphy K.E., Hodge M.R., Wu L., Butcher E.C. (2001). Unique subpopulations of CD56+ NK and NK-T peripheral blood lymphocytes identified by chemokine receptor expression repertoire. J. Immunol..

[40] Tokunaga R., Zhang W., Naseem M., Puccini A., Berger M.D., Soni S., McSkane M., Baba H., Lenz H.J. (2018). CXCL9, CXCL10, CXCL11/CXCR3 axis for immune activation—A target for novel cancer therapy. Cancer Treat. Rev..

[41] Russo E., Santoni A., Bernardini G. (2020). Tumor inhibition or tumor promotion? The duplicity of CXCR3 in cancer. J. Leukoc. Biol..

[42] Bronger H., Singer J., Windmuller C., Reuning U., Zech D., Delbridge C., Dorn J., Kiechle M., Schmalfeldt B., Schmitt M. (2016). CXCL9 and CXCL10 predict survival and are regulated by cyclooxygenase inhibition in advanced serous ovarian cancer. Br. J. Cancer.

[43] Kistner L., Doll D., Holtorf A., Nitsche U., Janssen K.P. (2017). Interferon-inducible CXC-chemokines are crucial immune modulators and survival predictors in colorectal cancer. Oncotarget.

[44] Cao Y., Huang H., Wang Z., Zhang G. (2017). The Inflammatory CXC Chemokines, GROalpha(high), IP-10(low), and MIG(low), in Tumor Microenvironment Can Be Used as New Indicators for Non-small Cell Lung Cancer Progression. Immunol. Investig..

[45] Sato Y., Motoyama S., Nanjo H., Wakita A., Yoshino K., Sasaki T., Nagaki Y., Liu J., Imai K., Saito H. (2016). CXCL10 Expression Status is Prognostic in Patients with Advanced Thoracic Esophageal Squamous Cell Carcinoma. Ann. Surg. Oncol..

[46] Bachelerie F., Ben-Baruch A., Burkhardt A.M., Combadiere C., Farber J.M., Graham G.J., Horuk R., Sparre-Ulrich A.H., Locati M., Luster A.D. (2014). International Union of Basic and Clinical Pharmacology. [corrected]. LXXXIX. Update on the extended family of chemokine receptors and introducing a new nomenclature for atypical chemokine receptors. Pharmacol. Rev..

[47] Ohmori Y., Schreiber R.D., Hamilton T.A. (1997). Synergy between interferon-gamma and tumor necrosis factor-alpha in transcriptional activation is mediated by cooperation between signal transducer and activator of transcription 1 and nuclear factor kappaB. J. Biol. Chem..

[48] Ohmori Y., Wyner L., Narumi S., Armstrong D., Stoler M., Hamilton T.A. (1993). Tumor necrosis factor-alpha induces cell type and tissue-specific expression of chemoattractant cytokines in vivo. Am. J. Pathol..

[49] Yoneyama H., Narumi S., Zhang Y., Murai M., Baggiolini M., Lanzavecchia A., Ichida T., Asakura H., Matsushima K. (2002). Pivotal role of dendritic cell-derived CXCL10 in the retention of T helper cell 1 lymphocytes in secondary lymph nodes. J. Exp. Med..

[50] de Mingo Pulido A., Gardner A., Hiebler S., Soliman H., Rugo H.S., Krummel M.F., Coussens L.M., Ruffell B. (2018). TIM-3 Regulates CD103(+) Dendritic Cell Function and Response to Chemotherapy in Breast Cancer. Cancer Cell.

[51] Plitas G., Konopacki C., Wu K., Bos P.D., Morrow M., Putintseva E.V., Chudakov D.M., Rudensky A.Y. (2016). Regulatory T Cells Exhibit Distinct Features in Human Breast Cancer. Immunity.

[52] Argyle D., Kitamura T. (2018). Targeting Macrophage-Recruiting Chemokines as a Novel Therapeutic Strategy to Prevent the Progression of Solid Tumors. Front. Immunol..

[53] Ueno T., Toi M., Saji H., Muta M., Bando H., Kuroi K., Koike M., Inadera H., Matsushima K. (2000). Significance of macrophage chemoattractant protein-1 in macrophage recruitment, angiogenesis, and survival in human breast cancer. Clin. Cancer Res..

[54] Sun H., Zhao L., Pan K., Zhang Z., Zhou M., Cao G. (2017). Integrated analysis of mRNA and miRNA expression profiles in pancreatic ductal adenocarcinoma. Oncol. Rep..

[55] Uhlen M., Zhang C., Lee S., Sjostedt E., Fagerberg L., Bidkhori G., Benfeitas R., Arif M., Liu Z., Edfors F. (2017). A pathology atlas of the human cancer transcriptome. Science.

[56] Uhlen M., Fagerberg L., Hallstrom B.M., Lindskog C., Oksvold P., Mardinoglu A., Sivertsson A., Kampf C., Sjostedt E., Asplund A. (2015). Proteomics. Tissue-based map of the human proteome. Science.

[57] Dangaj D., Bruand M., Grimm A.J., Ronet C., Barras D., Duttagupta P.A., Lanitis E., Duraiswamy J., Tanyi J.L., Benencia F. (2019). Cooperation between Constitutive and Inducible Chemokines Enables T Cell Engraftment and Immune Attack in Solid Tumors. Cancer Cell.

[58] Ruiz de Galarreta M., Bresnahan E., Molina-Sanchez P., Lindblad K.E., Maier B., Sia D., Puigvehi M., Miguela V., Casanova-Acebes M., Dhainaut M. (2019). beta-Catenin Activation Promotes Immune Escape and Resistance to Anti-PD-1 Therapy in Hepatocellular Carcinoma. Cancer Discov..

[59] Ventura-Clapier R., Garnier A., Veksler V. (2008). Transcriptional control of mitochondrial biogenesis: The central role of PGC-1alpha. Cardiovasc. Res..

[60] Derossi D.R., Amarante M.K., Guembarovski R.L., de Oliveira C.E.C., Suzuki K.M., Watanabe M.A.E., de Syllos Colus I.M. (2019). CCL5 protein level: Influence on breast cancer staging and lymph nodes commitment. Mol. Biol. Rep..

[61] Korbecki J., Kojder K., Siminska D., Bohatyrewicz R., Gutowska I., Chlubek D., Baranowska-Bosiacka I. (2020). CC Chemokines in a Tumor: A Review of Pro-Cancer and Anti-Cancer Properties of the Ligands of Receptors CCR1, CCR2, CCR3, and CCR4. Int. J. Mol. Sci..

[62] Li Y.Q., Liu F.F., Zhang X.M., Guo X.J., Ren M.J., Fu L. (2013). Tumor secretion of CCL22 activates intratumoral Treg infiltration and is independent prognostic predictor of breast cancer. PLoS ONE.

[63] Marshall L.A., Marubayashi S., Jorapur A., Jacobson S., Zibinsky M., Robles O., Hu D.X., Jackson J.J., Pookot D., Sanchez J. (2020). Tumors establish resistance to immunotherapy by regulating Treg recruitment via CCR4. J. Immunother. Cancer.

[64] Wang D., Yang L., Yu W., Wu Q., Lian J., Li F., Liu S., Li A., He Z., Liu J. (2019). Colorectal cancer cell-derived CCL20 recruits regulatory T cells to promote chemoresistance via FOXO1/CEBPB/NF-kappaB signaling. J. Immunother. Cancer.

[65] Zhang C.Y., Qi Y., Li X.N., Yang Y., Liu D.L., Zhao J., Zhu D.Y., Wu K., Zhou X.D., Zhao S. (2015). The role of CCL20/CCR6 axis in recruiting Treg cells to tumor sites of NSCLC patients. Biomed. Pharmacother..

[66] Lee J.J., Kao K.C., Chiu Y.L., Jung C.J., Liu C.J., Cheng S.J., Chang Y.L., Ko J.Y., Chia J.S. (2017). Enrichment of Human CCR6(+) Regulatory T Cells with Superior Suppressive Activity in Oral Cancer. J. Immunol..

[67] Liu J.Y., Li F., Wang L.P., Chen X.F., Wang D., Cao L., Ping Y., Zhao S., Li B., Thorne S.H. (2015). CTL- vs Treg lymphocyte-attracting chemokines, CCL4 and CCL20, are strong reciprocal predictive markers for survival of patients with oesophageal squamous cell carcinoma. Br. J. Cancer.

[68] Facciabene A., Peng X., Hagemann I.S., Balint K., Barchetti A., Wang L.P., Gimotty P.A., Gilks C.B., Lal P., Zhang L. (2011). Tumour hypoxia promotes tolerance and angiogenesis via CCL28 and T(reg) cells. Nature.

[69] Pham L.V., Bryant J.L., Mendez R., Chen J., Tamayo A.T., Xu-Monette Z.Y., Young K.H., Manyam G.C., Yang D., Medeiros L.J. (2016). Targeting the hexosamine biosynthetic pathway and O-linked N-acetylglucosamine cycling for therapeutic and imaging capabilities in diffuse large B-cell lymphoma. Oncotarget.

[70] OuYang L.Y., Wu X.J., Ye S.B., Zhang R.X., Li Z.L., Liao W., Pan Z.Z., Zheng L.M., Zhang X.S., Wang Z. (2015). Tumor-induced myeloid-derived suppressor cells promote tumor progression through oxidative metabolism in human colorectal cancer. J. Transl. Med..

[71] Ohta M., Tanaka F., Yamaguchi H., Sadanaga N., Inoue H., Mori M. (2005). The high expression of Fractalkine results in a better prognosis for colorectal cancer patients. Int. J. Oncol..

[72] Park M.H., Lee J.S., Yoon J.H. (2012). High expression of CX3CL1 by tumor cells correlates with a good prognosis and increased tumor-infiltrating CD8+ T cells, natural killer cells, and dendritic cells in breast carcinoma. J. Surg. Oncol..

[73] Liu J., Li Y., Zhu X., Li Q., Liang X., Xie J., Hu S., Peng W., Li C. (2019). Increased CX3CL1 mRNA expression level is a positive prognostic factor in patients with lung adenocarcinoma. Oncol. Lett..

[74] Marchica V., Toscani D., Corcione A., Bolzoni M., Storti P., Vescovini R., Ferretti E., Dalla Palma B., Vicario E., Accardi F. (2019). Bone Marrow CX3CL1/Fractalkine is a New Player of the Pro-Angiogenic Microenvironment in Multiple Myeloma Patients. Cancers (Basel).

[75] Korbecki J., Siminska D., Kojder K., Grochans S., Gutowska I., Chlubek D., Baranowska-Bosiacka I. (2020). Fractalkine/CX3CL1 in Neoplastic Processes. Int. J. Mol. Sci..

[76] Bottcher J.P., Bonavita E., Chakravarty P., Blees H., Cabeza-Cabrerizo M., Sammicheli S., Rogers N.C., Sahai E., Zelenay S., Reis e Sousa C. (2018). NK Cells Stimulate Recruitment of cDC1 into the Tumor Microenvironment Promoting Cancer Immune Control. Cell.

[77] Bodder J., Zahan T., van Slooten R., Schreibelt G., de Vries I.J.M., Florez-Grau G. (2020). Harnessing the cDC1-NK Cross-Talk in the Tumor Microenvironment to Battle Cancer. Front. Immunol..

[78] Bottcher J.P., Reis e Sousa C. (2018). The Role of Type 1 Conventional Dendritic Cells in Cancer Immunity. Trends Cancer.

[79] Murdoch C., Giannoudis A., Lewis C.E. (2004). Mechanisms regulating the recruitment of macrophages into hypoxic areas of tumors and other ischemic tissues. Blood.

[80] Zhang Y., Lv D., Kim H.J., Kurt R.A., Bu W., Li Y., Ma X. (2013). A novel role of hematopoietic CCL5 in promoting triple-negative mammary tumor progression by regulating generation of myeloid-derived suppressor cells. Cell Res..

[81] Nie Y., Huang H., Guo M., Chen J., Wu W., Li W., Xu X., Lin X., Fu W., Yao Y. (2019). Breast Phyllodes Tumors Recruit and Repolarize Tumor-Associated Macrophages via Secreting CCL5 to Promote Malignant Progression, Which Can Be Inhibited by CCR5 Inhibition Therapy. Clin. Cancer Res..

[82] Nishimura M., Umehara H., Nakayama T., Yoneda O., Hieshima K., Kakizaki M., Dohmae N., Yoshie O., Imai T. (2002). Dual functions of fractalkine/CX3C ligand 1 in trafficking of perforin+/granzyme B+ cytotoxic effector lymphocytes that are defined by CX3CR1 expression. J. Immunol..

[83] Umehara H., Bloom E.T., Okazaki T., Nagano Y., Yoshie O., Imai T. (2004). Fractalkine in vascular biology: From basic research to clinical disease. Arter. Thromb. Vasc. Biol..

[84] Rivas-Fuentes S., Salgado-Aguayo A., Arratia-Quijada J., Gorocica-Rosete P. (2021). Regulation and biological functions of the CX3CL1-CX3CR1 axis and its relevance in solid cancer: A mini-review. J. Cancer.

[85] Zlotnik A., Yoshie O. (2012). The chemokine superfamily revisited. Immunity.

[86] Tsou C.L., Haskell C.A., Charo I.F. (2001). Tumor necrosis factor-alpha-converting enzyme mediates the inducible cleavage of fractalkine. J. Biol. Chem..

[87] Hundhausen C., Misztela D., Berkhout T.A., Broadway N., Saftig P., Reiss K., Hartmann D., Fahrenholz F., Postina R., Matthews V. (2003). The disintegrin-like metalloproteinase ADAM10 is involved in constitutive cleavage of CX3CL1 (fractalkine) and regulates CX3CL1-mediated cell-cell adhesion. Blood.

[88] Bourd-Boittin K., Basset L., Bonnier D., L’Helgoualc’h A., Samson M., Theret N. (2009). CX3CL1/fractalkine shedding by human hepatic stellate cells: Contribution to chronic inflammation in the liver. J. Cell. Mol. Med..

[89] Tang J., Chen Y., Cui R., Li D., Xiao L., Lin P., Du Y., Sun H., Yu X., Zheng X. (2015). Upregulation of fractalkine contributes to the proliferative response of prostate cancer cells to hypoxia via promoting the G1/S phase transition. Mol. Med. Rep..

[90] Wang H., Cai J., Du S., Guo Z., Xin B., Wang J., Wei W., Shen X. (2017). Fractalkine/CX3CR1 induces apoptosis resistance and proliferation through the activation of the AKT/NF-kappaB cascade in pancreatic cancer cells. Cell Biochem. Funct..

[91] Nakayama T., Hirahara K., Onodera A., Endo Y., Hosokawa H., Shinoda K., Tumes D.J., Okamoto Y. (2017). Th2 Cells in Health and Disease. Annu. Rev. Immunol..

[92] Morel P.A., Oriss T.B. (1998). Crossregulation between Th1 and Th2 cells. Crit. Rev. Immunol..

[93] Yoshie O., Matsushima K. (2015). CCR4 and its ligands: From bench to bedside. Int. Immunol..

[94] Protti M.P., De Monte L. (2012). Cross-talk within the tumor microenvironment mediates Th2-type inflammation in pancreatic cancer. Oncoimmunology.

[95] Saravia J., Chapman N.M., Chi H. (2019). Helper T cell differentiation. Cell. Mol. Immunol..

[96] Basu A., Ramamoorthi G., Albert G., Gallen C., Beyer A., Snyder C., Koski G., Disis M.L., Czerniecki B.J., Kodumudi K. (2021). Differentiation and Regulation of TH Cells: A Balancing Act for Cancer Immunotherapy. Front. Immunol..

[97] Li Z., Jiang J., Wang Z., Zhang J., Xiao M., Wang C., Lu Y., Qin Z. (2008). Endogenous interleukin-4 promotes tumor development by increasing tumor cell resistance to apoptosis. Cancer Res..

[98] Lorvik K.B., Hammarstrom C., Fauskanger M., Haabeth O.A., Zangani M., Haraldsen G., Bogen B., Corthay A. (2016). Adoptive Transfer of Tumor-Specific Th2 Cells Eradicates Tumors by Triggering an In Situ Inflammatory Immune Response. Cancer Res..

[99] Dillon S.R., Sprecher C., Hammond A., Bilsborough J., Rosenfeld-Franklin M., Presnell S.R., Haugen H.S., Maurer M., Harder B., Johnston J. (2004). Interleukin 31, a cytokine produced by activated T cells, induces dermatitis in mice. Nat. Immunol..

[100] Kan T., Feldman E., Timaner M., Raviv Z., Shen-Orr S., Aronheim A., Shaked Y. (2020). IL-31 induces antitumor immunity in breast carcinoma. J. Immunother. Cancer.

[101] Bedoya S.K., Lam B., Lau K., Larkin J. (2013). Th17 cells in immunity and autoimmunity. Clin. Dev. Immunol..

[102] Knochelmann H.M., Dwyer C.J., Bailey S.R., Amaya S.M., Elston D.M., Mazza-McCrann J.M., Paulos C.M. (2018). When worlds collide: Th17 and Treg cells in cancer and autoimmunity. Cell. Mol. Immunol.

[103] Park H., Li Z., Yang X.O., Chang S.H., Nurieva R., Wang Y.H., Wang Y., Hood L., Zhu Z., Tian Q. (2005). A distinct lineage of CD4 T cells regulates tissue inflammation by producing interleukin 17. Nat. Immunol..

[104] Harrington L.E., Hatton R.D., Mangan P.R., Turner H., Murphy T.L., Murphy K.M., Weaver C.T. (2005). Interleukin 17-producing CD4+ effector T cells develop via a lineage distinct from the T helper type 1 and 2 lineages. Nat. Immunol..

[105] Brembilla N.C., Senra L., Boehncke W.H. (2018). The IL-17 Family of Cytokines in Psoriasis: IL-17A and Beyond. Front. Immunol..

[106] Dardalhon V., Korn T., Kuchroo V.K., Anderson A.C. (2008). Role of Th1 and Th17 cells in organ-specific autoimmunity. J. Autoimmun..

[107] Punt S., Langenhoff J.M., Putter H., Fleuren G.J., Gorter A., Jordanova E.S. (2015). The correlations between IL-17 vs. Th17 cells and cancer patient survival: A systematic review. Oncoimmunology.

[108] Bailey S.R., Nelson M.H., Himes R.A., Li Z., Mehrotra S., Paulos C.M. (2014). Th17 cells in cancer: The ultimate identity crisis. Front. Immunol..

[109] Zou W., Restifo N.P. (2010). T(H)17 cells in tumour immunity and immunotherapy. Nat. Rev. Immunol..

[110] Zhong W., Li Q. (2017). Rituximab or irradiation promotes IL-17 secretion and thereby induces resistance to rituximab or irradiation. Cell. Mol. Immunol..

[111] Kuang D.M., Peng C., Zhao Q., Wu Y., Zhu L.Y., Wang J., Yin X.Y., Li L., Zheng L. (2010). Tumor-activated monocytes promote expansion of IL-17-producing CD8+ T cells in hepatocellular carcinoma patients. J. Immunol..

[112] Housseau F., Wu S., Wick E.C., Fan H., Wu X., Llosa N.J., Smith K.N., Tam A., Ganguly S., Wanyiri J.W. (2016). Redundant Innate and Adaptive Sources of IL17 Production Drive Colon Tumorigenesis. Cancer Res..

[113] Tosolini M., Kirilovsky A., Mlecnik B., Fredriksen T., Mauger S., Bindea G., Berger A., Bruneval P., Fridman W.H., Pages F. (2011). Clinical impact of different classes of infiltrating T cytotoxic and helper cells (Th1, th2, treg, th17) in patients with colorectal cancer. Cancer Res..

[114] Yamada Y., Saito H., Ikeguchi M. (2012). Prevalence and clinical relevance of Th17 cells in patients with gastric cancer. J. Surg. Res..

[115] Zhang J.P., Yan J., Xu J., Pang X.H., Chen M.S., Li L., Wu C., Li S.P., Zheng L. (2009). Increased intratumoral IL-17-producing cells correlate with poor survival in hepatocellular carcinoma patients. J. Hepatol..

[116] Chen X., Wan J., Liu J., Xie W., Diao X., Xu J., Zhu B., Chen Z. (2010). Increased IL-17-producing cells correlate with poor survival and lymphangiogenesis in NSCLC patients. Lung Cancer.

[117] He S., Fei M., Wu Y., Zheng D., Wan D., Wang L., Li D. (2011). Distribution and clinical significance of Th17 cells in the tumor microenvironment and peripheral blood of pancreatic cancer patients. Int. J. Mol. Sci..

[118] Numasaki M., Fukushi J., Ono M., Narula S.K., Zavodny P.J., Kudo T., Robbins P.D., Tahara H., Lotze M.T. (2003). Interleukin-17 promotes angiogenesis and tumor growth. Blood.

[119] Numasaki M., Watanabe M., Suzuki T., Takahashi H., Nakamura A., McAllister F., Hishinuma T., Goto J., Lotze M.T., Kolls J.K. (2005). IL-17 enhances the net angiogenic activity and in vivo growth of human non-small cell lung cancer in SCID mice through promoting CXCR-2-dependent angiogenesis. J. Immunol..

[120] Chang S.H., Mirabolfathinejad S.G., Katta H., Cumpian A.M., Gong L., Caetano M.S., Moghaddam S.J., Dong C. (2014). T helper 17 cells play a critical pathogenic role in lung cancer. Proc. Natl. Acad. Sci. USA.

[121] He D., Li H., Yusuf N., Elmets C.A., Li J., Mountz J.D., Xu H. (2010). IL-17 promotes tumor development through the induction of tumor promoting microenvironments at tumor sites and myeloid-derived suppressor cells. J. Immunol..

[122] Kehlen A., Thiele K., Riemann D., Rainov N., Langner J. (1999). Interleukin-17 stimulates the expression of IkappaB alpha mRNA and the secretion of IL-6 and IL-8 in glioblastoma cell lines. J. Neuroimmunol..

[123] Zhou Y., Toh M.L., Zrioual S., Miossec P. (2007). IL-17A versus IL-17F induced intracellular signal transduction pathways and modulation by IL-17RA and IL-17RC RNA interference in AGS gastric adenocarcinoma cells. Cytokine.

[124] Najafi S., Mirshafiey A. (2019). The role of T helper 17 and regulatory T cells in tumor microenvironment. Immunopharmacol. Immunotoxicol..

[125] Ohta A., Kini R., Ohta A., Subramanian M., Madasu M., Sitkovsky M. (2012). The development and immunosuppressive functions of CD4(+) CD25(+) FoxP3(+) regulatory T cells are under influence of the adenosine-A2A adenosine receptor pathway. Front. Immunol..

[126] Acosta-Rodriguez E.V., Rivino L., Geginat J., Jarrossay D., Gattorno M., Lanzavecchia A., Sallusto F., Napolitani G. (2007). Surface phenotype and antigenic specificity of human interleukin 17-producing T helper memory cells. Nat. Immunol..

[127] Kadomoto S., Izumi K., Mizokami A. (2020). The CCL20-CCR6 Axis in Cancer Progression. Int. J. Mol. Sci..

[128] Chen D., Jiang R., Mao C., Shi L., Wang S., Yu L., Hu Q., Dai D., Xu H. (2012). Chemokine/chemokine receptor interactions contribute to the accumulation of Th17 cells in patients with esophageal squamous cell carcinoma. Hum. Immunol..

[129] Yang X.W., Jiang H.X., Lei R., Lu W.S., Tan S.H., Qin S.Y. (2018). Recruitment and significance of Th22 cells and Th17 cells in malignant ascites. Oncol. Lett..

[130] Yang L., Qi Y., Hu J., Tang L., Zhao S., Shan B. (2012). Expression of Th17 cells in breast cancer tissue and its association with clinical parameters. Cell Biochem. Biophys..

[131] Alves J.J.P., De Medeiros Fernandes T.A.A., De Araujo J.M.G., Cobucci R.N.O., Lanza D.C.F., Bezerra F.L., Andrade V.S., Fernandes J.V. (2018). Th17 response in patients with cervical cancer. Oncol. Lett..

[132] Lv L., Pan K., Li X.D., She K.L., Zhao J.J., Wang W., Chen J.G., Chen Y.B., Yun J.P., Xia J.C. (2011). The accumulation and prognosis value of tumor infiltrating IL-17 producing cells in esophageal squamous cell carcinoma. PLoS ONE.

[133] Ye Z.J., Zhou Q., Gu Y.Y., Qin S.M., Ma W.L., Xin J.B., Tao X.N., Shi H.Z. (2010). Generation and differentiation of IL-17-producing CD4+ T cells in malignant pleural effusion. J. Immunol..

[134] Zhang Y.L., Li J., Mo H.Y., Qiu F., Zheng L.M., Qian C.N., Zeng Y.X. (2010). Different subsets of tumor infiltrating lymphocytes correlate with NPC progression in different ways. Mol. Cancer.

[135] Miyahara Y., Odunsi K., Chen W., Peng G., Matsuzaki J., Wang R.F. (2008). Generation and regulation of human CD4+ IL-17-producing T cells in ovarian cancer. Proc. Natl. Acad. Sci. USA.

[136] Sfanos K.S., Bruno T.C., Maris C.H., Xu L., Thoburn C.J., DeMarzo A.M., Meeker A.K., Isaacs W.B., Drake C.G. (2008). Phenotypic analysis of prostate-infiltrating lymphocytes reveals TH17 and Treg skewing. Clin. Cancer Res..

[137] Huang Y., Wang J., Jia P., Li X., Pei G., Wang C., Fang X., Zhao Z., Cai Z., Yi X. (2019). Clonal architectures predict clinical outcome in clear cell renal cell carcinoma. Nat. Commun..

[138] Koyama K., Kagamu H., Miura S., Hiura T., Miyabayashi T., Itoh R., Kuriyama H., Tanaka H., Tanaka J., Yoshizawa H. (2008). Reciprocal CD4+ T-cell balance of effector CD62Llow CD4+ and CD62LhighCD25+ CD4+ regulatory T cells in small cell lung cancer reflects disease stage. Clin. Cancer Res..

[139] Kryczek I., Banerjee M., Cheng P., Vatan L., Szeliga W., Wei S., Huang E., Finlayson E., Simeone D., Welling T.H. (2009). Phenotype, distribution, generation, and functional and clinical relevance of Th17 cells in the human tumor environments. Blood.

[140] Kryczek I., Wei S., Szeliga W., Vatan L., Zou W. (2009). Endogenous IL-17 contributes to reduced tumor growth and metastasis. Blood.

[141] Martin-Orozco N., Muranski P., Chung Y., Yang X.O., Yamazaki T., Lu S., Hwu P., Restifo N.P., Overwijk W.W., Dong C. (2009). T helper 17 cells promote cytotoxic T cell activation in tumor immunity. Immunity.

[142] Ankathatti Munegowda M., Deng Y., Mulligan S.J., Xiang J. (2011). Th17 and Th17-stimulated CD8(+) T cells play a distinct role in Th17-induced preventive and therapeutic antitumor immunity. Cancer Immunol. Immunother..

[143] Benchetrit F., Ciree A., Vives V., Warnier G., Gey A., Sautes-Fridman C., Fossiez F., Haicheur N., Fridman W.H., Tartour E. (2002). Interleukin-17 inhibits tumor cell growth by means of a T-cell-dependent mechanism. Blood.

[144] Al Omar S., Flanagan B.F., Almehmadi M., Christmas S.E. (2013). The effects of IL-17 upon human natural killer cells. Cytokine.

[145] Jovanovic D.V., Di Battista J.A., Martel-Pelletier J., Jolicoeur F.C., He Y., Zhang M., Mineau F., Pelletier J.P. (1998). IL-17 stimulates the production and expression of proinflammatory cytokines, IL-beta and TNF-alpha, by human macrophages. J. Immunol..

[146] Lu L., Pan K., Zheng H.X., Li J.J., Qiu H.J., Zhao J.J., Weng D.S., Pan Q.Z., Wang D.D., Jiang S.S. (2013). IL-17A promotes immune cell recruitment in human esophageal cancers and the infiltrating dendritic cells represent a positive prognostic marker for patient survival. J. Immunother..

[147] Tong Z., Yang X.O., Yan H., Liu W., Niu X., Shi Y., Fang W., Xiong B., Wan Y., Dong C. (2012). A protective role by interleukin-17F in colon tumorigenesis. PLoS ONE.

[148] Weber G.F., Gaertner F.C., Erl W., Janssen K.P., Blechert B., Holzmann B., Weighardt H., Essler M. (2006). IL-22-mediated tumor growth reduction correlates with inhibition of ERK1/2 and AKT phosphorylation and induction of cell cycle arrest in the G2-M phase. J. Immunol..

[149] Castermans K., Tabruyn S.P., Zeng R., van Beijnum J.R., Eppolito C., Leonard W.J., Shrikant P.A., Griffioen A.W. (2008). Angiostatic activity of the antitumor cytokine interleukin-21. Blood.

[150] Furuta S., Jeng Y.M., Zhou L., Huang L., Kuhn I., Bissell M.J., Lee W.H. (2011). IL-25 causes apoptosis of IL-25R-expressing breast cancer cells without toxicity to nonmalignant cells. Sci. Transl. Med..

[151] Josefowicz S.Z., Lu L.F., Rudensky A.Y. (2012). Regulatory T cells: Mechanisms of differentiation and function. Annu. Rev. Immunol..

[152] Tanaka A., Sakaguchi S. (2017). Regulatory T cells in cancer immunotherapy. Cell Res..

[153] Li C., Jiang P., Wei S., Xu X., Wang J. (2020). Regulatory T cells in tumor microenvironment: New mechanisms, potential therapeutic strategies and future prospects. Mol. Cancer.

[154] Sawant D.V., Yano H., Chikina M., Zhang Q., Liao M., Liu C., Callahan D.J., Sun Z., Sun T., Tabib T. (2019). Adaptive plasticity of IL-10(+) and IL-35(+) Treg cells cooperatively promotes tumor T cell exhaustion. Nat. Immunol..

[155] Walker L.S., Sansom D.M. (2011). The emerging role of CTLA4 as a cell-extrinsic regulator of T cell responses. Nat. Rev. Immunol..

[156] Liang B., Workman C., Lee J., Chew C., Dale B.M., Colonna L., Flores M., Li N., Schweighoffer E., Greenberg S. (2008). Regulatory T cells inhibit dendritic cells by lymphocyte activation gene-3 engagement of MHC class II. J. Immunol..

[157] Maruhashi T., Okazaki I.M., Sugiura D., Takahashi S., Maeda T.K., Shimizu K., Okazaki T. (2018). LAG-3 inhibits the activation of CD4(+) T cells that recognize stable pMHCII through its conformation-dependent recognition of pMHCII. Nat. Immunol..

[158] Ohta A., Sitkovsky M. (2001). Role of G-protein-coupled adenosine receptors in downregulation of inflammation and protection from tissue damage. Nature.

[159] Lee C.R., Kwak Y., Yang T., Han J.H., Park S.H., Ye M.B., Lee W., Sim K.Y., Kang J.A., Kim Y.C. (2016). Myeloid-Derived Suppressor Cells Are Controlled by Regulatory T Cells via TGF-beta during Murine Colitis. Cell Rep..

[160] Ghiringhelli F., Puig P.E., Roux S., Parcellier A., Schmitt E., Solary E., Kroemer G., Martin F., Chauffert B., Zitvogel L. (2005). Tumor cells convert immature myeloid dendritic cells into TGF-beta-secreting cells inducing CD4+CD25+ regulatory T cell proliferation. J. Exp. Med..

[161] Ohue Y., Nishikawa H. (2019). Regulatory T (Treg) cells in cancer: Can Treg cells be a new therapeutic target?. Cancer Sci..

[162] Mizukami Y., Kono K., Kawaguchi Y., Akaike H., Kamimura K., Sugai H., Fujii H. (2008). CCL17 and CCL22 chemokines within tumor microenvironment are related to accumulation of Foxp3+ regulatory T cells in gastric cancer. Int. J. Cancer.

[163] Liu W., Wei X., Li L., Wu X., Yan J., Yang H., Song F. (2017). CCR4 mediated chemotaxis of regulatory T cells suppress the activation of T cells and NK cells via TGF-beta pathway in human non-small cell lung cancer. Biochem. Biophys. Res. Commun..

[164] Tsujikawa T., Yaguchi T., Ohmura G., Ohta S., Kobayashi A., Kawamura N., Fujita T., Nakano H., Shimada T., Takahashi T. (2013). Autocrine and paracrine loops between cancer cells and macrophages promote lymph node metastasis via CCR4/CCL22 in head and neck squamous cell carcinoma. Int. J. Cancer.

[165] Wertel I., Surowka J., Polak G., Barczynski B., Bednarek W., Jakubowicz-Gil J., Bojarska-Junak A., Kotarski J. (2015). Macrophage-derived chemokine CCL22 and regulatory T cells in ovarian cancer patients. Tumour Biol..

[166] Zhang W., Chen L., Ma K., Zhao Y., Liu X., Wang Y., Liu M., Liang S., Zhu H., Xu N. (2016). Polarization of macrophages in the tumor microenvironment is influenced by EGFR signaling within colon cancer cells. Oncotarget.

[167] Nakayama T., Hieshima K., Nagakubo D., Sato E., Nakayama M., Kawa K., Yoshie O. (2004). Selective induction of Th2-attracting chemokines CCL17 and CCL22 in human B cells by latent membrane protein 1 of Epstein-Barr virus. J. Virol..

[168] Takegawa S., Jin Z., Nakayama T., Oyama T., Hieshima K., Nagakubo D., Shirakawa A.K., Tsuzuki T., Nakamura S., Yoshie O. (2008). Expression of CCL17 and CCL22 by latent membrane protein 1-positive tumor cells in age-related Epstein-Barr virus-associated B-cell lymphoproliferative disorder. Cancer Sci..

[169] Zhou S.L., Zhou Z.J., Hu Z.Q., Huang X.W., Wang Z., Chen E.B., Fan J., Cao Y., Dai Z., Zhou J. (2016). Tumor-Associated Neutrophils Recruit Macrophages and T-Regulatory Cells to Promote Progression of Hepatocellular Carcinoma and Resistance to Sorafenib. Gastroenterology.

[170] Mishalian I., Bayuh R., Eruslanov E., Michaeli J., Levy L., Zolotarov L., Singhal S., Albelda S.M., Granot Z., Fridlender Z.G. (2014). Neutrophils recruit regulatory T-cells into tumors via secretion of CCL17--a new mechanism of impaired antitumor immunity. Int. J. Cancer.

[171] Omland S.H., Wettergren E.E., Mollerup S., Asplund M., Mourier T., Hansen A.J., Gniadecki R. (2017). Cancer associated fibroblasts (CAFs) are activated in cutaneous basal cell carcinoma and in the peritumoural skin. BMC Cancer.

[172] Kimura S., Nanbu U., Noguchi H., Harada Y., Kumamoto K., Sasaguri Y., Nakayama T. (2019). Macrophage CCL22 expression in the tumor microenvironment and implications for survival in patients with squamous cell carcinoma of the tongue. J. Oral Pathol. Med..

[173] Yoshie O. (2021). CCR4 as a Therapeutic Target for Cancer Immunotherapy. Cancers (Basel).

[174] Sugiyama D., Nishikawa H., Maeda Y., Nishioka M., Tanemura A., Katayama I., Ezoe S., Kanakura Y., Sato E., Fukumori Y. (2013). Anti-CCR4 mAb selectively depletes effector-type FoxP3+CD4+ regulatory T cells, evoking antitumor immune responses in humans. Proc. Natl. Acad. Sci. USA.

[175] Robles O., Jackson J.J., Marshall L., Talay O., Chian D., Cutler G., Diokno R., Hu D.X., Jacobson S., Karbarz E. (2020). Novel Piperidinyl-Azetidines as Potent and Selective CCR4 Antagonists Elicit Antitumor Response as a Single Agent and in Combination with Checkpoint Inhibitors. J. Med. Chem.

[176] Yamamoto S., Matsuo K., Nagakubo D., Higashiyama S., Nishiwaki K., Oiso N., Kawada A., Yoshie O., Nakayama T. (2018). A CCR4 antagonist enhances DC activation and homing to the regional lymph node and shows potent vaccine adjuvant activity through the inhibition of regulatory T-cell recruitment. J. Pharmacol. Sci..

[177] Piseddu I., Rohrle N., Knott M.M.L., Moder S., Eiber S., Schnell K., Vetter V., Meyer B., Layritz P., Kuhnemuth B. (2020). Constitutive Expression of CCL22 Is Mediated by T Cell-Derived GM-CSF. J. Immunol..

[178] Sallusto F., Palermo B., Lenig D., Miettinen M., Matikainen S., Julkunen I., Forster R., Burgstahler R., Lipp M., Lanzavecchia A. (1999). Distinct patterns and kinetics of chemokine production regulate dendritic cell function. Eur. J. Immunol..

[179] Hirahara K., Liu L., Clark R.A., Yamanaka K., Fuhlbrigge R.C., Kupper T.S. (2006). The majority of human peripheral blood CD4+CD25highFoxp3+ regulatory T cells bear functional skin-homing receptors. J. Immunol..

[180] Caligiuri A., Pastore M., Lori G., Raggi C., Di Maira G., Marra F., Gentilini A. (2020). Role of Chemokines in the Biology of Cholangiocarcinoma. Cancers (Basel).

[181] Beider K., Abraham M., Begin M., Wald H., Weiss I.D., Wald O., Pikarsky E., Abramovitch R., Zeira E., Galun E. (2009). Interaction between CXCR4 and CCL20 pathways regulates tumor growth. PLoS ONE.

[182] Cinier J., Hubert M., Besson L., Di Roio A., Rodriguez C., Lombardi V., Caux C., Menetrier-Caux C. (2021). Recruitment and Expansion of Tregs Cells in the Tumor Environment-How to Target Them?. Cancers (Basel).

[183] Soler D., Chapman T.R., Poisson L.R., Wang L., Cote-Sierra J., Ryan M., McDonald A., Badola S., Fedyk E., Coyle A.J. (2006). CCR8 expression identifies CD4 memory T cells enriched for FOXP3+ regulatory and Th2 effector lymphocytes. J. Immunol..

[184] Iellem A., Mariani M., Lang R., Recalde H., Panina-Bordignon P., Sinigaglia F., D’Ambrosio D. (2001). Unique chemotactic response profile and specific expression of chemokine receptors CCR4 and CCR8 by CD4(+)CD25(+) regulatory T cells. J. Exp. Med..

[185] Barsheshet Y., Wildbaum G., Levy E., Vitenshtein A., Akinseye C., Griggs J., Lira S.A., Karin N. (2017). CCR8(+)FOXp3(+) Treg cells as master drivers of immune regulation. Proc. Natl. Acad. Sci. USA.

[186] De Vlaeminck Y., Gonzalez-Rascon A., Goyvaerts C., Breckpot K. (2016). Cancer-Associated Myeloid Regulatory Cells. Front. Immunol..

[187] Malekghasemi S., Majidi J., Baghbanzadeh A., Abdolalizadeh J., Baradaran B., Aghebati-Maleki L. (2020). Tumor-Associated Macrophages: Protumoral Macrophages in Inflammatory Tumor Microenvironment. Adv. Pharm. Bull..

[188] Heusinkveld M., van der Burg S.H. (2011). Identification and manipulation of tumor associated macrophages in human cancers. J. Transl. Med..

[189] Bingle L., Brown N.J., Lewis C.E. (2002). The role of tumour-associated macrophages in tumour progression: Implications for new anticancer therapies. J. Pathol..

[190] Kumar V., Patel S., Tcyganov E., Gabrilovich D.I. (2016). The Nature of Myeloid-Derived Suppressor Cells in the Tumor Microenvironment. Trends Immunol..

[191] Youn J.I., Gabrilovich D.I. (2010). The biology of myeloid-derived suppressor cells: The blessing and the curse of morphological and functional heterogeneity. Eur. J. Immunol..

[192] Qian B.Z., Li J., Zhang H., Kitamura T., Zhang J., Campion L.R., Kaiser E.A., Snyder L.A., Pollard J.W. (2011). CCL2 recruits inflammatory monocytes to facilitate breast-tumour metastasis. Nature.

[193] Toh B., Wang X., Keeble J., Sim W.J., Khoo K., Wong W.C., Kato M., Prevost-Blondel A., Thiery J.P., Abastado J.P. (2011). Mesenchymal transition and dissemination of cancer cells is driven by myeloid-derived suppressor cells infiltrating the primary tumor. PLoS Biol..

[194] Karin N. (2020). The Development and Homing of Myeloid-Derived Suppressor Cells: From a Two-Stage Model to a Multistep Narrative. Front. Immunol..

[195] Lecot P., Sarabi M., Pereira Abrantes M., Mussard J., Koenderman L., Caux C., Bendriss-Vermare N., Michallet M.C. (2019). Neutrophil Heterogeneity in Cancer: From Biology to Therapies. Front. Immunol..

[196] Cheng Y., Mo F., Li Q., Han X., Shi H., Chen S., Wei Y., Wei X. (2021). Targeting CXCR2 inhibits the progression of lung cancer and promotes therapeutic effect of cisplatin. Mol. Cancer.

[197] Miao M., De Clercq E., Li G. (2020). Clinical significance of chemokine receptor antagonists. Expert Opin. Drug Metab. Toxicol..

[198] Nagarsheth N., Wicha M.S., Zou W. (2017). Chemokines in the cancer microenvironment and their relevance in cancer immunotherapy. Nat. Rev. Immunol..

[199] Mollica Poeta V., Massara M., Capucetti A., Bonecchi R. (2019). Chemokines and Chemokine Receptors: New Targets for Cancer Immunotherapy. Front. Immunol..

[200] Flores-Toro J.A., Luo D., Gopinath A., Sarkisian M.R., Campbell J.J., Charo I.F., Singh R., Schall T.J., Datta M., Jain R.K. (2020). CCR2 inhibition reduces tumor myeloid cells and unmasks a checkpoint inhibitor effect to slow progression of resistant murine gliomas. Proc. Natl. Acad. Sci. USA.

[201] Yang Z., Li H., Wang W., Zhang J., Jia S., Wang J., Wei J., Lei D., Hu K., Yang X. (2019). CCL2/CCR2 Axis Promotes the Progression of Salivary Adenoid Cystic Carcinoma via Recruiting and Reprogramming the Tumor-Associated Macrophages. Front. Oncol..

[202] Maeda S., Murakami K., Inoue A., Yonezawa T., Matsuki N. (2019). CCR4 Blockade Depletes Regulatory T Cells and Prolongs Survival in a Canine Model of Bladder Cancer. Cancer Immunol. Res..

[203] Blattner C., Fleming V., Weber R., Himmelhan B., Altevogt P., Gebhardt C., Schulze T.J., Razon H., Hawila E., Wildbaum G. (2018). CCR5(+) Myeloid-Derived Suppressor Cells Are Enriched and Activated in Melanoma Lesions. Cancer Res..

[204] Dépis F., Hu C., Weaver J., McGrath L., Klebanov B., Buggé J., Umiker B., Fregeau C., Upadhyay D., Singh A. (2020). Abstract 4532:Preclinical Evaluation of JTX-1811, an Anti-CCR8 Antibody with Enhanced ADCC Activity, for Preferential Depletion of Tumor-Infiltrating Regulatory T Cells. Cancer Res..

[205] Lake A.,  Warren M., Das S., Wells C., Scrivens M., Smith E., Palombella V., Etemad-Gilbertson B., Holland P., Dulak A. (2020). 726 SRF114 Is a Fully Human, CCR8 Selective IgG1 Antibody That Induces Destruction of Tumor Tregs through ADCC. J. Immunother. Cancer.

[206] Lu S., Hu C., Gan X., Wang Y., Zhao C., Ding Y., He J., Du Q., Lv X., Qin B. (2020). 711 HBM1022, a Novel Anti-CCR8 Antibody Depletes Tumor-Infiltrating Regulatory T Cells via Enhanced ADCC Activity, Mediates Potent Anti-Tumor Activity with Keytruda. J. Immunother. Cancer.

[207] Rankin A., Naik E. (2020). 861 Development of FPA157, an Anti-CCR8 Depleting Antibody Engineered to Preferentially Eliminate Tumor-Infiltrating T Regulatory Cells. J. Immunother. Cancer.

[208] Campbell J.R., McDonald B.R., Mesko P.B., Siemers N.O., Singh P.B., Selby M., Sproul T.W., Korman A.J., Vlach L.M., Houser J. (2021). Fc-Optimized Anti-CCR8 Antibody Depletes Regulatory T Cells in Human Tumor Models. Cancer Res..

[209] Noel M., O’Reilly E.M., Wolpin B.M., Ryan D.P., Bullock A.J., Britten C.D., Linehan D.C., Belt B.A., Gamelin E.C., Ganguly B. (2020). Phase 1b study of a small molecule antagonist of human chemokine (C-C motif) receptor 2 (PF-04136309) in combination with nab-paclitaxel/gemcitabine in first-line treatment of metastatic pancreatic ductal adenocarcinoma. Invest. New Drugs.

[210] Cohen E.E.W., Pishvaian M.J., Shepard D.R., Wang D., Weiss J., Johnson M.L., Chung C.H., Chen Y., Huang B., Davis C.B. (2019). A phase Ib study of utomilumab (PF-05082566) in combination with mogamulizumab in patients with advanced solid tumors. J. Immunother. Cancer.

[211] Zamarin D., Hamid O., Nayak-Kapoor A., Sahebjam S., Sznol M., Collaku A., Fox F.E., Marshall M.A., Hong D.S. (2020). Mogamulizumab in Combination with Durvalumab or Tremelimumab in Patients with Advanced Solid Tumors: A Phase I Study. Clin. Cancer Res..

[212] Doi T., Muro K., Ishii H., Kato T., Tsushima T., Takenoyama M., Oizumi S., Gemmoto K., Suna H., Enokitani K. (2019). A Phase I Study of the Anti-CC Chemokine Receptor 4 Antibody, Mogamulizumab, in Combination with Nivolumab in Patients with Advanced or Metastatic Solid Tumors. Clin. Cancer Res..

[213] Biasci D., Smoragiewicz M., Connell C.M., Wang Z., Gao Y., Thaventhiran J.E.D., Basu B., Magiera L., Johnson T.I., Bax L. (2020). CXCR4 inhibition in human pancreatic and colorectal cancers induces an integrated immune response. Proc. Natl. Acad. Sci. USA.

[214] Bockorny B., Semenisty V., Macarulla T., Borazanci E., Wolpin B.M., Stemmer S.M., Golan T., Geva R., Borad M.J., Pedersen K.S. (2020). BL-8040, a CXCR4 antagonist, in combination with pembrolizumab and chemotherapy for pancreatic cancer: The COMBAT trial. Nat. Med..

[215] Pernas S., Martin M., Kaufman P.A., Gil-Martin M., Gomez Pardo P., Lopez-Tarruella S., Manso L., Ciruelos E., Perez-Fidalgo J.A., Hernando C. (2018). Balixafortide plus eribulin in HER2-negative metastatic breast cancer: A phase 1, single-arm, dose-escalation trial. Lancet Oncol..

[216] O’Hara M.H., Messersmith W., Kindler H., Zhang W., Pitou C., Szpurka A.M., Wang D., Peng S.B., Vangerow B., Khan A.A. (2020). Safety and Pharmacokinetics of CXCR4 Peptide Antagonist, LY2510924, in Combination with Durvalumab in Advanced Refractory Solid Tumors. J. Pancreat. Cancer.

[217] de Mingo Pulido A., Hanggi K., Celias D.P., Gardner A., Li J., Batista-Bittencourt B., Mohamed E., Trillo-Tinoco J., Osunmakinde O., Pena R. (2021). The inhibitory receptor TIM-3 limits activation of the cGAS-STING pathway in intra-tumoral dendritic cells by suppressing extracellular DNA uptake. Immunity.

[218] Ji R.R., Chasalow S.D., Wang L., Hamid O., Schmidt H., Cogswell J., Alaparthy S., Berman D., Jure-Kunkel M., Siemers N.O. (2012). An immune-active tumor microenvironment favors clinical response to ipilimumab. Cancer Immunol. Immunother..

[219] Hannesdottir L., Tymoszuk P., Parajuli N., Wasmer M.H., Philipp S., Daschil N., Datta S., Koller J.B., Tripp C.H., Stoitzner P. (2013). Lapatinib and doxorubicin enhance the Stat1-dependent antitumor immune response. Eur J. Immunol..

[220] Hong M., Puaux A.L., Huang C., Loumagne L., Tow C., Mackay C., Kato M., Prevost-Blondel A., Avril M.F., Nardin A. (2011). Chemotherapy induces intratumoral expression of chemokines in cutaneous melanoma, favoring T-cell infiltration and tumor control. Cancer Res..

[221] Berlato C., Khan M.N., Schioppa T., Thompson R., Maniati E., Montfort A., Jangani M., Canosa M., Kulbe H., Hagemann U.B. (2017). A CCR4 antagonist reverses the tumor-promoting microenvironment of renal cancer. J. Clin. Investig..

[222] Kohli K., Pillarisetty V.G., Kim T.S. (2021). Key chemokines direct migration of immune cells in solid tumors. Cancer Gene Ther..

[223] Whiteside S.K., Grant F.M., Gyori D.S., Conti A.G., Imianowski C.J., Kuo P., Nasrallah R., Sadiyah F., Lira S.A., Tacke F. (2021). CCR8 marks highly suppressive Treg cells within tumours but is dispensable for their accumulation and suppressive function. Immunology.

[224] Katoh H., Wang D., Daikoku T., Sun H., Dey S.K., Dubois R.N. (2013). CXCR2-expressing myeloid-derived suppressor cells are essential to promote colitis-associated tumorigenesis. Cancer Cell.

[225] Steele C.W., Karim S.A., Leach J.D.G., Bailey P., Upstill-Goddard R., Rishi L., Foth M., Bryson S., McDaid K., Wilson Z. (2016). CXCR2 Inhibition Profoundly Suppresses Metastases and Augments Immunotherapy in Pancreatic Ductal Adenocarcinoma. Cancer Cell.

[226] Groth C., Arpinati L., Shaul M.E., Winkler N., Diester K., Gengenbacher N., Weber R., Arkhypov I., Lasser S., Petrova V. (2021). Blocking Migration of Polymorphonuclear Myeloid-Derived Suppressor Cells Inhibits Mouse Melanoma Progression. Cancers (Basel).

[227] Jurcevic S., Humfrey C., Uddin M., Warrington S., Larsson B., Keen C. (2015). The effect of a selective CXCR2 antagonist (AZD5069) on human blood neutrophil count and innate immune functions. Br. J. Clin. Pharmacol..

[228] Li X., Yao W., Yuan Y., Chen P., Li B., Li J., Chu R., Song H., Xie D., Jiang X. (2017). Targeting of tumour-infiltrating macrophages via CCL2/CCR2 signalling as a therapeutic strategy against hepatocellular carcinoma. Gut.

[229] Deshmane S.L., Kremlev S., Amini S., Sawaya B.E. (2009). Monocyte chemoattractant protein-1 (MCP-1): An overview. J. Interf. Cytokine Res..

[230] Jiao X., Nawab O., Patel T., Kossenkov A.V., Halama N., Jaeger D., Pestell R.G. (2019). Recent Advances Targeting CCR5 for Cancer and Its Role in Immuno-Oncology. Cancer Res..

[231] Kuloglu E.S., McCaslin D.R., Markley J.L., Volkman B.F. (2002). Structural rearrangement of human lymphotactin, a C chemokine, under physiological solution conditions. J. Biol. Chem..

[232] Matsuo K., Kitahata K., Kawabata F., Kamei M., Hara Y., Takamura S., Oiso N., Kawada A., Yoshie O., Nakayama T. (2018). A Highly Active Form of XCL1/Lymphotactin Functions as an Effective Adjuvant to Recruit Cross-Presenting Dendritic Cells for Induction of Effector and Memory CD8(+) T Cells. Front. Immunol..

[233] Kamei M., Matsuo K., Imanishi H., Hara Y., Quen Y.S., Kamiyama F., Oiso N., Kawada A., Okada N., Nakayama T. (2020). Transcutaneous immunization with a highly active form of XCL1 as a vaccine adjuvant using a hydrophilic gel patch elicits long-term CD8(+) T cell responses. J. Pharmacol. Sci..

[234] Morein D., Erlichman N., Ben-Baruch A. (2020). Beyond Cell Motility: The Expanding Roles of Chemokines and Their Receptors in Malignancy. Front. Immunol..

[235] Keeley E.C., Mehrad B., Strieter R.M. (2008). Chemokines as mediators of neovascularization. Arter. Thromb. Vasc. Biol..

